# Single‐Cell Mitochondrial Lineage Tracing Decodes Fate Decision and Spatial Clonal Architecture in Human Hematopoietic Organoids

**DOI:** 10.1002/advs.202518084

**Published:** 2026-01-21

**Authors:** Yan Xue, Junhao Su, Yiming Chao, Lu Liu, Xinyi Lin, Yang Xiang, Mun Kay Ho, Zezhuo Su, Junyi Chen, Zhuojuan Luo, Chengqi Lin, Ruibang Luo, Theo Aurich, Jianfeng Wu, Kelvin Sin Chi Cheung, Yuanhua Huang, Joshua W. K. Ho, Ryohichi Sugimura

**Affiliations:** ^1^ School of Biomedical Sciences Li Ka Shing Faculty of Medicine The University of Hong Kong Hong Kong SAR China; ^2^ Laboratory of Data Discovery For Health Limited (D^2^4H) Hong Kong Science Park Hong Kong Hong Kong; ^3^ Inno HK Centre For Translational Stem Cell Biology Hong Kong China; ^4^ Department of Orthopaedics and Traumatology School of Clinical Medicine Li Ka Shing Faculty of Medicine The University of Hong Kong Hong Kong SAR China; ^5^ Jiangsu Provincial Key Laboratory of Critical Care Medicine Southeast University Nanjing China; ^6^ Jiangsu Province Hi‐Tech Key Laboratory For Biomedical Research Southeast University Nanjing China; ^7^ Department of Computer Science School of Computing and Data Science The University of Hong Kong Hong Kong SAR China; ^8^ Heidelberg University Hospital Germany; ^9^ Department of Diagnostic Radiology School of Clinical Medicine Li Ka Shing Faculty of Medicine The University of Hong Kong Hong Kong SAR China; ^10^ Department of Statistics and Actuarial Science Faculty of Science The University of Hong Kong Hong Kong SAR China; ^11^ School of Biomedical Engineering The University of Hong Kong Hong Kong SAR China

**Keywords:** cell fate decisions, hematopoietic organoids, mitochondrial DNA variant, single‐cell lineage tracing, spatial transcriptomics

## Abstract

Lineage tracing at single‐cell resolution is vital for mapping cell fate decisions, yet synthetic barcoding faces limitations in precision, diversity, and toxicity—especially in human pluripotent stem cells (hPSCs). Here, we repurpose naturally occurring somatic mutations in mitochondrial transcripts from single‐cell RNA sequencing as endogenous genetic barcodes. By enriching mitochondrial reads and applying a robust computational pipeline, we identified clonally informative variants to trace hematopoietic lineage emergence from hPSCs during early embryogenesis. Integrating mitochondrial barcoding with synthetic lineage tracing, we modeled embryonic tissue development and reconstructed the transcriptional logic and regulatory networks driving fate specification using a dynamical systems model. Extending this approach to spatial transcriptomics, we mapped the clonal architecture of human embryonic organoids, revealing spatial zonation orchestrated by NOTCH‐mediated crosstalk between stromal cells and hematopoietic progenitors. This multimodal strategy links clonal dynamics with niche‐driven fate decisions, offering a scalable, non‐invasive method to dissect tissue organization in development and disease. Together, our work establishes a scalable, non‐invasive multimodal framework that leverages endogenous mitochondrial DNA variants to reconstruct high‐resolution spatiotemporal clonal dynamics and decode niche‐driven fate decisions in a human stem cell‐derived model. This approach provides a powerful strategy for dissecting tissue self‐organization in development and disease.

## Graphical abstract

This study repurposes mitochondrial DNA mutations as endogenous barcodes for lineage tracing in human pluripotent stem cell‐derived organoids. Integrated with transcriptomic and spatial data, it reveals NOTCH‐mediated stromal‐progenitor crosstalk orchestrates clonal dynamics and spatial zonation during early hematopoietic development, offering a non‐invasive method to dissect tissue organization.

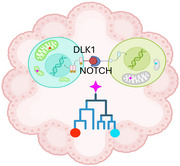

## 1 Introduction

Tracing the progeny of individual cells is essential for understanding cell‐division dynamics and fate decisions during embryonic development, stem cell differentiation, and disease progression. Prospective lineage tracing systems have been widely applied to study embryogenesis, hematopoiesis, neural development, and cancer biology [1, 2, 3]. Integrating genetic barcodes with single‐cell transcriptomics enables simultaneous lineage reconstruction and state characterization [4, 5]. However, technical limitations—including barcode homoplasy, restricted diversity, and detection sensitivity—compromise lineage resolution and accuracy. Current synthetic barcoding systems, such as Lineage And RNA RecoverY (LARRY), face inherent trade‐offs: while enabling high‐throughput clonal tracking through expressed DNA tags, they generate artifacts like spurious multi‐progenitor labelling [6]. Furthermore, dependency on multi‐locus genetic modifications risks perturbing native cell behaviours—sustained CRISPR‐Cas9 activity, for instance, can induce cytotoxicity in human pluripotent stem cells [7]. Such limitations underscore an unmet need for non‐invasive, high‐fidelity lineage recorders.

Retrospective lineage tracing, leveraging endogenous somatic mutations, offers a compelling alternative by eliminating artificial manipulation. Mitochondrial DNA (mtDNA) mutations are particularly advantageous due to their high mutation rate, heteroplasmic segregation, and compatibility with single‐cell assays [8, 9]. Recent advances in mitochondrial variant enrichment (MAESTER) and identification algorithms (MQuad) now enable precise lineage tracing at scale [10, 11]. Yet, the effectiveness of mitochondrial variants as lineage tracing markers to characterize the spatial arrangement of cell states and phenotypic transition hasn't been explored.

Spatial transcriptomics has significantly enhanced our understanding of developmental hierarchies, cellular plasticity, and diverse tissue microenvironmentsQ in both healthy and diseased tissues [12]. The proper functioning of tissues relies on intricate interactions between cells, creating complex and dynamic cellular ecosystems [13]. Since cells often have limited mobility within tissues, their physical proximity often indicates relatedness, and their differentiation can be influenced by signals from neighbouring cells and morphogenetic gradients [14]. Tissues maintain stability during development through the coordinated efforts of various cell types, each with specialized roles and functions. Studying the spatial context of cell‐fate decision‐making can aid in distinguishing between intrinsic and extrinsic factors that impact cell differentiation and developmental processes [15, 16, 17]. Current spatial profiling can also capture substantial auxiliary information that influences cell fate, lineage, and differentiation [18, 19, 20, 21].

Here, we leveraged naturally occurring mitochondrial somatic mutations from single‐cell RNA‐seq as endogenous genetic barcodes for lineage tracing in human pluripotent stem cell (hPSC)‐derived embryonic organoids (HEMOs). By integrating this approach with synthetic barcoding, we reconstructed embryonic tissue development and delineated early cell fate choices. Furthermore, coupling mitochondrial clonal tracking with spatial transcriptomics revealed the coordinated emergence of hematopoietic cells and their supportive niche cells from common progenitors within yolk sac‐like tissues. We further demonstrated that this spatially restricted clonal architecture is shaped by NOTCH‐mediated stromal‐progenitor crosstalk. Together, our findings establish mitochondrial variants as a powerful tool for resolving high‐resolution spatial clonal dynamics in human developmental models.

## 2 Results

### 2.1 Distinct Early Fate Choices and Trajectories Uncovered by Lineage Tracing in Hematopoietic Organoids

**FIGURE 1:**
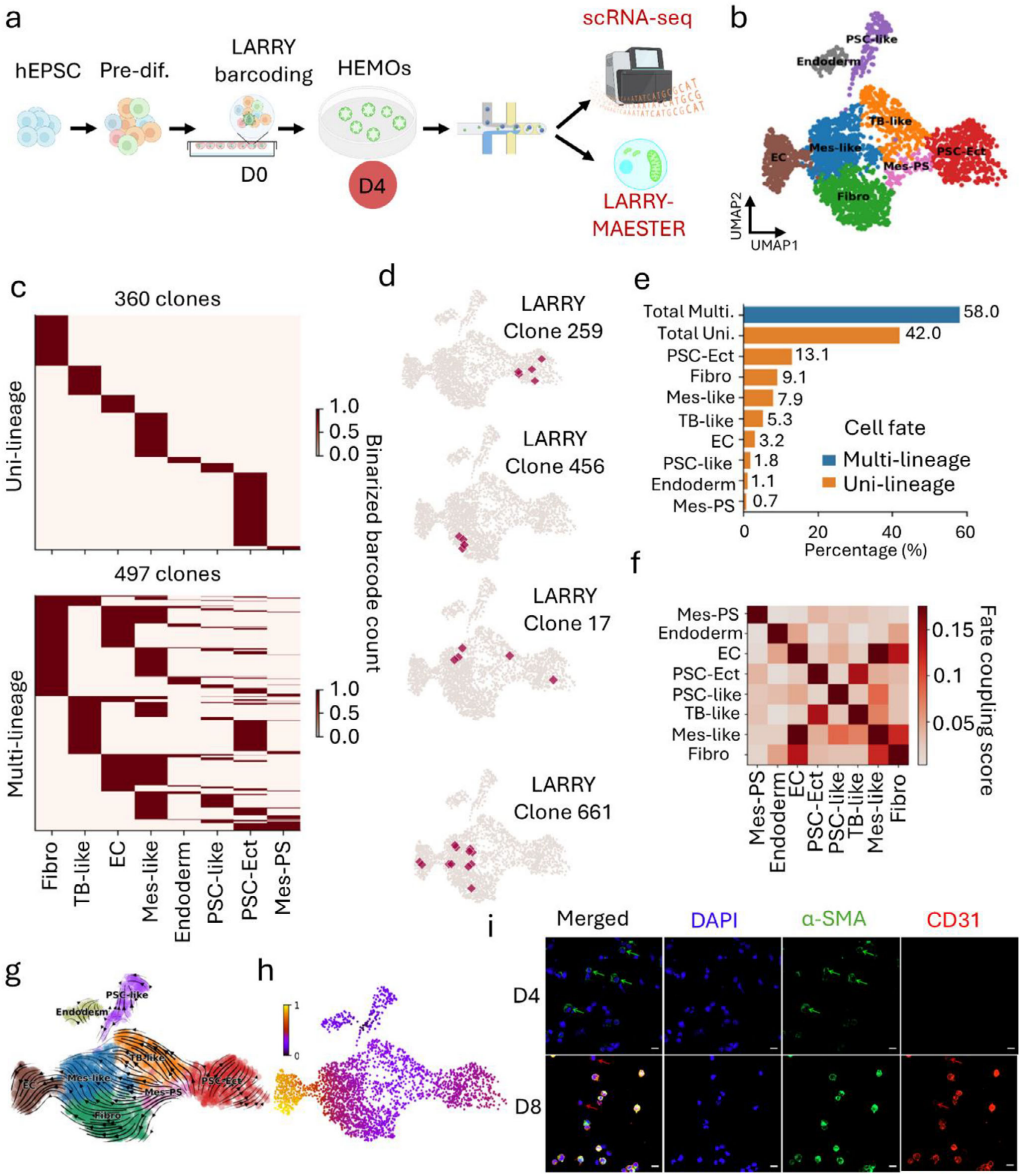
Lineage tracing reveals distinct cell fates during the early development of HEMOs (D4). (a) Overall study design for clonal tracing of early hematopoietic lineages. Pre‐differentiated (Pre‐diff.) human expanded pluripotent stem cells (hEPSC) were barcoded using the Lineage And RNA RecoverY (LARRY) system on Day 0 (D0). Barcoded human embryonic hematopoietic organoids (HEMOs) were harvested at Day 4 (D4) and subjected to single‐cell RNA sequencing (scRNA‐seq) and mitochondrial variant enrichment (MAESTER) to generate LARRY‐MAESTER libraries for lineage reconstruction. (b) UMAP plot illustrates the transition of pluripotent cells into three distinct germ layers (ectoderm, mesoderm, endoderm) and specialized lineages. Key cell types include pluripotent stem cells‐like (PSC‐like), pluripotent stem cells with ectoderm specification (PSC‐Ect), trophoblast‐like cells (TB‐like), mesoderm primitive streak/cardiac mesoderm‐like cells (Mes‐PS), mesoderm‐like cells (Mes‐like), endothelial cells (EC), and fibroblasts (Fibro). (c) Heatmaps showing clonal fate predictions generated by CoSpar. Each row represents a LARRY clone, and each column corresponds to a specific lineage. (d) UMAP plots showing the example clones with uni‐ (top two) or multi‐lineage (bottom two) characteristics. (e) Bar chart showing the proportion of clones in uni‐ and multi‐lineages. (f) Cell fate coupling revealed by CoSpar. The heatmap highlights coupling between Mes‐like and EC as well as Fibro. (g) RNA velocity field describes the fate decisions of major HEMO lineages in LARRY dataset. The velocity field is projected onto a PCA plot with arrows indicating the local average velocity evaluated on a regular grid. RNA velocity was estimated without cell or gene pooling. (h) Inferred pseudotime trajectory across the cellular continuum. Color intensity reflects pseudotime progression (scale: 0 = early, 1 = late). (i) Immunofluorescence analysis of fibroblasts and endothelial cells at Day 4 (D4) and Day 8 (D8). Mesenchymal cells were FACS‐sorted based on PDGFRα expression via flow cytometry at Day 2 then cultured. Subsequent immunofluorescence staining was performed to identify Fibro and EC using α‐SMA and CD31 at D4 (D2+2 days culture) and D8 (D2+6 days culture) respectively. Shown are representative images from three independent experiments (*n* = 3). The green and red arrows denote single‐positive cells for α‐SMA and CD31, respectively. Scale bar = 20 µm.

A significant challenge in stem cell haematopoiesis lies in linking molecular variations within progenitor cells to their ability to produce mature cell types. In this study, we employed expressed DNA barcodes to track transcriptomes clonally over time, applying this method to investigate hematopoietic clonal dynamics in HEMO. We infected M1‐hPSCs with LARRY, which employs a barcoding system detectable by single‐cell RNA sequencing (scRNA‐seq) for clonal labelling, to investigate hematopoiesis within our HEMOs (Figure 1a). Our aim is to track the dynamic process of blood regeneration in the human embryonic stem cell‐derived organoid model, where hematopoietic stem and progenitor cells give rise to various blood cell lineages. Due to the 3D structure of the organoid, it is challenging for us to separate half of the cell population as the “early stage” and allow the remaining half to undergo further divisions to become “late stage” cells. Instead, we barcoded two batches of stem cells and harvested the D4 and D8 organoids, respectively, to generate early and late stage cells. We excluded the minimal number of overlapping barcodes (4 out of 7,361), ensuring that the barcode repertoires were non‐overlapping between experiments. Consequently, all lineage tracing analyses were confined within each time point, enabling the reconstruction of stage‐specific clonal relationships without cross‐temporal assumptions. This design allows us to compare early and late fate decisions as captured by independent snapshots of organoid development.

To ensure that the comparisons between D4 and D8 reflect genuine biological transitions rather than technical variations introduced by processing in separate batches, we first performed an integrated analysis using Harmony, a batch‐correction algorithm. This integration confirmed that cells of the same annotated type robustly clustered together regardless of their batch origin (Figure S1), validating our core cell type definitions. Reassured that major batch effects were mitigated, we proceeded to analyze the D4 and D8 datasets separately for all subsequent in‐depth analyses to preserve and resolve the distinct, stage‐specific biological complexity of each time point.

At the early stage of HEMO hematopoiesis (D4), we captured a diverse cell population, including endoderm, pluripotent stem cells‐like (PSC‐like), pluripotent stem cells with ectoderm specification (PSC‐Ect), trophoblast‐like cells (TB‐like), mesoderm primitive streak/cardiac (Mes‐PS), mesoderm‐like cells (Mes‐like), endothelial cells (EC), and fibroblasts (Fibro) (Figure 1b and Figure S2a). This suggests that our HEMO recapitulates key developmental processes, where pluripotent cells transition into three distinct germ layers (ectoderm, mesoderm, endoderm) and specialized lineages. The strong intra‐cell‐type connectedness [22] not only validated our clustering results but also provided additional evidence for the dynamic differentiation continuum within the HEMO system (Figure S2b). LARRY barcoded a total of 4659 cells, grouping them into 2453 clones. We noted that 65% of the LARRY clones are one‐cell clones (Figure S2c), aligning with the expected distribution of clone size [4]. After filtering out the one‐cell clones, the remaining cells were assigned to 857 clones based on distinct LARRY barcodes (Figure S2d). The clone sizes ranged from 2 to 15 cells. Notably, 58% of LARRY clones (481 clones) are two‐cell clones. The number of large clones was relatively small, with only 15 clones (2%) containing 10 or more cells (Figure S2d).

CoSpar analysis [6] revealed distinct cell fate in LARRY barcoded clones (Figure 1c‐f). Among the LARRY clones analysed, 42% (360 clones) were lineage specific, encompassing endoderm, PSC‐like, Mes‐PS, PSC‐Ect, TB‐like, and Fibro lineages (Figure 1c, d). These uni‐lineage clones, such as LARRY Clone 259 and 456, are spatially confined to a distinct region with the UMAP embedding (Figure 1d, top panel), suggesting that they exhibit distinct lineage commitments and differentiation potentials. Notably, a significant proportion of LARRY clones spanned multiple lineages (Figure 1c–e), displaying divergent spatial distributions in the UMAP embeddings (Figure 1d, bottom panel). For instance, LARRY Clone 17 gave rise to PSC‐Ect, Mes‐like, and TB‐like cells, and Clone 661 produced EC, Mes‐like, and Fibro cells (Figure 1d, bottom), demonstrating the multipotency of their progenitors and revealing inherent biases toward specific fate combinations.

Our lineage coupling analysis identified non‐random interactions. Mes‐like exhibited the strongest coupling with EC and Fibro lineages (Figure 1f). Consistently, the dynamics of gene expression as captured by RNA velocity revealed pronounced directional flows converging toward each lineage in early HEMOs (Figure 1g). The reconstructed velocity field mapped fate decisions across the principal lineages. Mes‐like derivatives exhibited a strong primary bias toward EC specification (Figure 1g). These dynamic patterns aligned with the clonal relationships in Figure 1f. Furthermore, Mes‐PS populations showed a dominant differentiation trend toward the Fibro lineage (Figure 1g). Pseudotime ordering positions the Mes‐like lineage as a progenitor state within a differentiation continuum that culminates in the acquisition of a mature EC identity (Figure 1h).

We next sought to experimentally validate the differentiation trajectories of PDGFRα^+^ mesenchymal progenitors predicted by our computational analyses. To this end, we isolated PDGFRα^+^ cells via flow cytometry at Day 2 and performed time‐course immunofluorescence analysis at Day 4 and Day 8 to trace their fate using markers for fibroblasts (α‐SMA) and endothelial cells (CD31). This approach revealed a clear temporal sequence of lineage specification (Figure 1i). At D4, we readily identified α‐SMA^+^ fibroblasts (green arrows, Figure 1i), whereas CD31^+^ endothelial cells were virtually absent. By contrast, by D8, a distinct population of CD31^+^ endothelial cells had emerged within the mesenchymal progenitor pool (red arrows, Figure 1i). Our live imaging further captured the bipotent differentiation of mesenchymal progenitors into fibroblasts and endothelial cells (Movie S1). The sequential appearance of fibroblasts followed by endothelial cells provides experimental evidence consistent with the RNA velocity and pseudotime predictions (Figure 1g,h); together, these data depict the Mes‐like lineage as a progenitor state progressing toward a mature endothelial cell identity. These findings collectively underscore the pivotal role of PDGFRα^+^ mesenchymal progenitors in orchestrating early cell fate decisions by sequentially generating multiple lineages.

### 2.2 Gene Module Dynamics Decode the Hierarchical Roadmap of Fate Decisions

To move beyond static correlations and reconstruct the dynamic regulatory logic underpinning cell fate specification in HEMOs, we employed cell2fate [23]. This framework deconstructs the transcriptome into a series of co‐regulated gene expression modules, the sequential activation and repression of which define a continuous trajectory from progenitors to mature lineages. Unlike standard clustering, these modules represent core transcriptional programs with inferred dynamics, offering a mechanistic view of the differentiation process. Our goal was to identify these fundamental programs and determine if they provide a direct explanatory framework for the non‐random lineage couplings we observed through our empirical clonal tracing.

**FIGURE 2:**
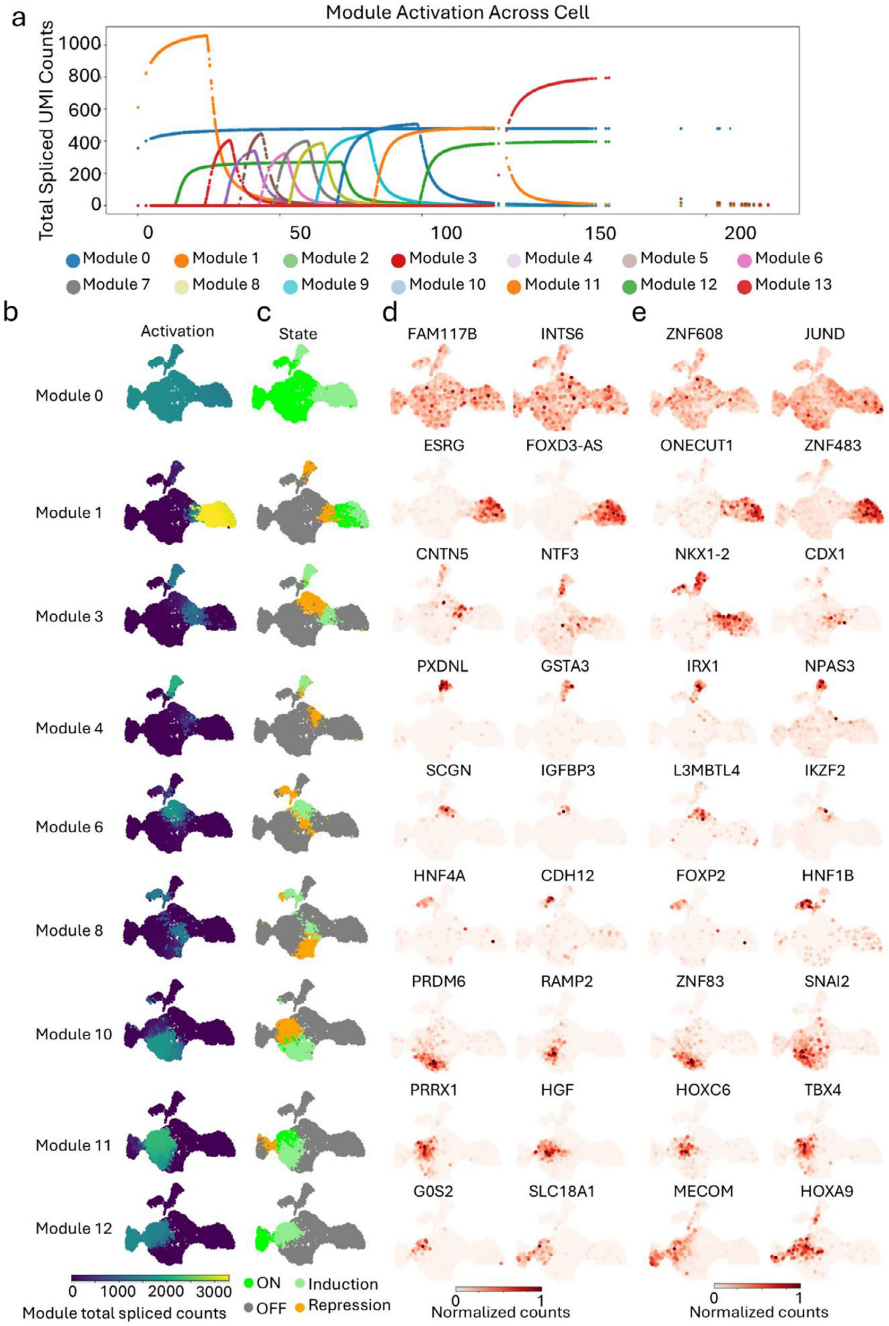
Module decomposition of cell2fate uncover coupled lineage specification in a human hematopoietic organoid. (a) Total spliced mRNA abundance attributable to each cell2fate module across pseudotime. (b) Module activity scores per cell, quantified as the fraction of spliced counts explained by each module. (c) Expression patterns of key modules across the hematopoietic organoid lineage spectrum. (d) Top two marker genes for each module, identified based on the proportion of their transcription rate attributed to the module. (e) Key regulatory transcription factors (TFs) within each module, defined as module marker genes with known TF activity.

The initial state of the system was defined by Module 0, which was ubiquitously active across all early cell types (Figure 2a–c). This module was characterized by high expression of marker genes FAM117B and INTS6, and driven by transcription factors ZNF608 and JUND, indicative of a primordial, proliferative, and metabolically active foundation from which all subsequent lineages emerge (Figure 2d,e). Functional enrichment analysis confirmed this foundational role, showing significant enrichment for broad, essential processes such as RNA Polymerase II Transcription and Gene Expression (Figure S3).

Module 1 was specifically activated in the PSC‐Ect population. This state was characterized by residual expression of naive pluripotency markers such as ESRG and ZNF483, alongside early lineage‐priming factors including FOXD3‐AS1 and ONECUT1. This molecular signature, coupled with its strong enrichment for Anterior/Posterior Pattern Specification (Figure S3), indicates that the PSC‐Ect state represents a lineage‐primed but uncommitted progenitor poised to respond to early embryonic patterning cues, while retaining broad developmental potential, rather than a definitively specified ectodermal lineage.

Module 3 became specifically active in both Mes‐PS and a distinct pluripotent (PSC‐like) population. This module was characterized by marker genes CNTN5 and NTF3, and regulated by transcription factors NKX1‐2 and CDX1 (Figure 2b–e). Functional enrichment analysis revealed that Module 3 is dominated by broad transcriptional regulatory programs—including Regulation of DNA‐templated Transcription—alongside organ‐specific developmental terms such as Kidney Development and Endodermal Cell Fate Commitment. (Figure S3). Notably, the enrichment for TFAP2 family regulation further underscores its association with multipotency, as AP‐2 factors are known regulators of diverse ectodermal, mesodermal, and endodermal derivatives (Figure S3). These findings support a model wherein Module 3 marks a transitional state in which pluripotent cells, having downregulated ectodermal programs (Module 1), activate a global transcriptional machinery poised for lineage diversification across multiple germ layers prior to full commitment.

Subsequently, Module 4 expression became restricted solely to the PSC‐like population, with loss of Mes‐PS activity. This shift was marked by the expression of genes PXDNL and GSTA3, under the regulatory control of IRX1 and NPAS3, indicating a refinement of the pluripotent state, potentially toward a more specialized or transient progenitor identity. This refined state was uniquely characterized by a dual functional signature: enrichment for Transcriptional Regulation of Pluripotent Stem Cells, coupled with a specific enrichment for Fat Cell Differentiation, hinting at a biased potential toward a mesodermal stromal fate (Figure S3).

Later stages of differentiation were driven by the activation of lineage‐specific modules. Module 6 was uniquely associated with TB‐like cells, defined by markers SCGN and IGFBP3, and controlled by the factors L3MBTL4 and IKZF2 (Figure 2b–e). Module 8 activation specifically heralded endoderm specification, evidenced by the expression of the classic marker HNF4A alongside CDH12, and directed by the key endoderm‐regulatory transcription factors FOXP2 and HNF1B (Figure 2b–e).

Further lineage diversification was marked by the activation of Module 10, which drove the emergence of a Fibro identity. This state was characterized by genes PRDM6 and RAMP2, and transcription factors ZNF83 and SNAI2, the latter indicative of potential roles in epithelial‐to‐mesenchymal transition (Figure 2b–e). Concurrently, Module 11 activation specified Mes‐like cells, marked by PRRX1 and HGF expression and governed by the regulators HOXC6 and TBX4. Strikingly, Module 11 was defined by a highly significant enrichment for a suite of precise organogenesis terms, including those governing Cardiac Right Ventricle Morphogenesis and Aortic Valve Development (Figure S3). This signature suggests that the Mes‐like population possesses a broad organ‐generative transcriptional program, characteristic of a multipotent central progenitor, rather than being restricted to a single lineage (Figure S3).

Finally, the acquisition of an EC fate was propelled by Module 12, defined by the expression of G0S2 and SLC18A1 and orchestrated by the transcription factors MECOM and HOXA9 (Figure 2b–e). Mechanistically, Module 12's role was illuminated not by generic endothelial markers, but by its top enrichment for Smooth Muscle Cell Differentiation and Regulation of Myeloid Cell Differentiation, suggesting a differentiation process involving a transitional progenitor state with dual smooth muscle and hematopoietic features (Figure S3). Consistent with this model of hematopoietic potential, Module 13 was specifically and highly expressed in a subset of mature EC and showed significant enrichment for gene sets related to myeloid cell differentiation and hematopoiesis (Figure S3). These molecular signatures are consistent with those reported in hemogenic endothelium, positioning this EC subset as the functional realization of the bipotent potential inferred from Module 12.

Collectively, this modular series of gene expression activations and restrictions outlines a hierarchical roadmap of cell fate decisions within the HEMO. While the principle of hierarchical differentiation is well‐established, our model decodes its specific transcriptional implementation: from broad, foundational and patterning programs (Modules 0, 1), through primed and refined multipotent states (Modules 3, 4), to the execution of precise, lineage‐specific organogenesis and differentiation programs (Modules 6, 8, 10‐13) that collectively build the hematopoietic niche.

Having established this hierarchical roadmap of regulatory modules, we sought to directly test whether they provide a mechanistic basis for the non‐random lineage couplings we uncovered through our empirical clonal tracing (Figure 1c–f). Strikingly, the module dynamics offered a clear explanatory framework for these key clonal observations.

Specifically, our lineage coupling analysis had identified non‐random interactions, with the Mes‐like lineage exhibiting the strongest coupling to both EC and Fibro lineages (Figure 1f). This bifurcated potential was elegantly explained by the distinct activities of module activities of Modules 10, 11, and 12. Module 11 was strongly activated in Mes‐like cells, marking them as a central progenitor state. From this state, the specific activation of Module 10 in Fibro (and its repression in Mes‐like) drove stromal specification, while the specific activation of Module 12 in EC (with clear induction in Mes‐like) highlighted the privileged differentiation path from mesenchymal progenitors to endothelium (Figure 2). This modular logic—a shared progenitor module (11) giving way to mutually exclusive effector modules (10 vs. 12)—directly provides a transcriptional rationale for the cell‐autonomous fate biases we observed clonally, moving beyond a simple description of hierarchy to reveal its underlying regulatory architecture.

These modular patterns were consistent with RNA velocity trajectories, which revealed pronounced directional flows from Mes‐like toward EC specification (Figure 1h), corroborating the role of Modules 12 and 13 in endothelial commitment (Figure 2 and Figure S3e). The strong bias of Mes‐PS toward Fibro was also reinforced by Module 10‐specific activation. Pseudotime analysis positioned the Mes‐like population as a central progenitor within a continuous lineage continuum culminating in EC identity (Figure 1h), aligning with the transient co‐expression patterns in Modules 11 and 12 during fate restriction (Figure 2).

Collectively, these multi‐modal data integrate regulatory modules with fate dynamics, providing a coherent model of early lineage diversification in which modular gene activation and repression direct bifurcated trajectories from multipotent mesenchymal progenitors to stromal and endothelial fates. This modular logic underpins the cell‐autonomous fate biases observed clonally, offering a transcriptional rationale for fate decisions that may appear stochastic from single‐timepoint transcriptomes.

### 2.3 Clonal Expansion and Restricted Fate Potential Characterize Late‐Stage Organoid Maturation

At D8, HEMOs transitioned into a more developmentally advanced and stable state, characterized by clearly segregated lineage compartments and the emergence of novel progenitor populations, including erythro‐myeloid progenitors (EMP) and cardiac mesoderm‐like cells (CM‐like) (Figure S4a, b). The predominance of EMPs and the absence of lymphoid lineages in our HEMO model are consistent with the profile of very early embryonic hematopoiesis, prior to the emergence of definitive HSCs [4]. Analysis of the D8 LARRY dataset (2,698 cells assigned to 1,391 clones) revealed that 73% of clones (1026/1,391) were one‐cell clones (Figure S4c, d). After excluding these singletons, the remaining 1,578 cells clustered into 347 clones (Figure S4e), indicating reduced clonal diversity as hematopoiesis progressed. Notably, late‐stage HEMO exhibited significant clonal expansion: the largest clone contained 29 cells—double the maximum observed in early stage—and 10% of clones (38/347) exceeded 10 cells, a marked increase compared to earlier timepoints (Figure S4e). This shift suggests the stabilization and dominance of specific clones during later developmental stages. Additionally, we identified lineage‐specific LARRY clones at D8, including PSC‐like, CM‐like, PSC‐Ect, ectoderm‐like cells (Ect‐like), endoderm, and EMP lineage‐specific clones (Figure S5a). We also observed 60% LARRY clones were multi‐lineages (Figure S5b). Similarly, these uni‐lineage clones, such as LARRY Clone 185 and 188, are spatially confined to a distinct region with the UMAP embedding (Figure S5c, top panel), suggesting that they exhibit distinct lineage commitments and differentiation potentials. The multi‐lineage LARRY clones displayed divergent spatial distributions in the UMAP embeddings (Figure S5c, bottom panel). For instance, LARRY Clone 71 gave rise to Fibro and EC cells (Figure S5c, bottom), demonstrating the multipotency of their progenitors.

Lineage coupling analysis revealed a significant reduction in broad non‐random interactions compared to D4, reflecting enhanced lineage restriction and specialization (Figure S5d). Despite this overall segregation, strong coupling between EC and Fibro lineages persisted (Figure S5d), reaffirming a conserved bifurcated differentiation path toward vascular and stromal fates—a hallmark of multipotent mesenchymal progenitor activity. Notably, a moderate but distinct clonal coupling was detected between EMP and EC lineages (Figure S5d). This interaction suggests a developmental kinship or shared progenitor trajectory between endothelial and erythro‐myeloid lineages, supporting the notion of hemogenic endothelial‐like activity or a vascular‐affiliated origin for a subset of EMPs within the HEMO model.

RNA velocity analysis further corroborated these findings, showing refined and pronounced directional flows toward well‐defined terminal lineages (Figure S5e). The reconstructed velocity fields illustrated a maturation of fate decisions: the EC lineage demonstrated heightened maturity and structural formation (Figure S5e). In contrast to its strong EC‐specification bias at D4, the Mes‐like population had largely shifted its differentiation trajectory by D8, with only a minor subset of cells retaining potential to contribute to the Fibro lineage (Figure S5e). Concurrently, PSC‐Ect lineages showed committed flow toward ectodermal fates (Figure S5e). This supports the interpretation that earlier pluripotent or bipotent states have resolved into more restricted identities by D8. The pseudotime trajectory positions EC as a late‐stage population, aligning with their phenotypic maturation into functional EC (Figure S5f). Together, these results indicate that D8 represents a stage of increased lineage resolution and structural maturity within HEMOs. The persistence of EC–Fibro coupling—alongside the emerging EMP–EC relationship and the clear separation of other lineages—highlights the sustained role of mesenchymal and endothelial progenitors in supporting hematopoietic and stromal crosstalk even amid overall developmental progression toward compartmentalization.

### 2.4 Benchmarking of mtDNA Variant Calling Pipelines Establishes MQuad for Reliable Clonal Inference

At the early stage (D4), our LARRY analysis revealed a high frequency of two‐cell clones (58%, Figure S2d). While this could, in part, reflect transcriptional similarity between recently divided sister cells, we sought to determine if the observed fate combinations were biologically meaningful. We found that the distribution of two‐cell fate pairs was non‐random. Notably, the most frequent cross‐lineage pairs were Mes‐like+ EC and Mes‐like+ Fibro (Figure 1e,f). This specific combination is indicative of a fundamental developmental bifurcation, as the lateral plate mesoderm in vivo is the established precursor for both cardiac (endothelial) and limb bud (stromal) lineages [24]. To conclusively distinguish this biologically meaningful clonal multilineage potential from mere technical associations or sister‐cell effects, we required an orthogonal lineage‐tracing method.

To this end, we integrated mtDNA mutations as naturally occurring, endogenous somatic markers for lineage tracing. mtDNA mutations accumulate stochastically during cell division and are clonally inherited, making them ideal orthogonal markers to validate synthetic barcode‐based clonal groupings. Unlike engineered barcodes, mtDNA variants are not subject to homoplasy from viral integration bias and provide an independent clonal record. We implemented the MAESTER protocol to delve deeply into mtDNA for resolving clonal substructures, allowing simultaneous assessment of transcriptome and lineage history without additional experimental burden.

**FIGURE 3:**
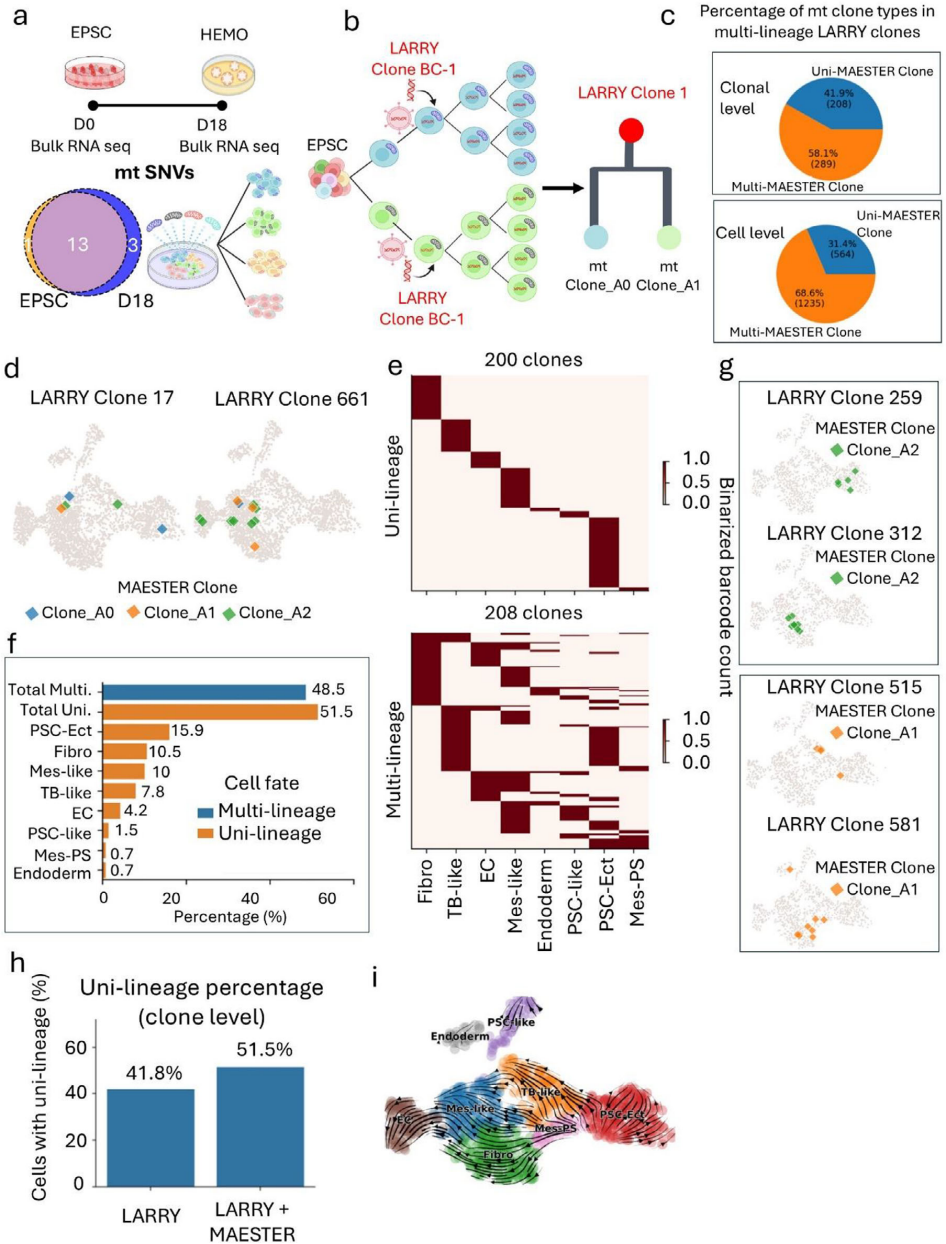
Mitochondrial variant barcoding resolves LARRY noise and uncovers cell fate decisions during early development of HEMOs (D4). (a) Bulk RNA‐seq analysis reveals substantial overlap in mtDNA variants between EPSCs (D0) and D18 HEMOs, supporting their utility as stable lineage markers. (b) Diagram shows integration of mtDNA variant barcoding can address LARRY barcode homoplasy, enhancing clonal resolution and lineage tracing accuracy. (c) The pie charts show a comparison of the relative abundance of uni‐ and multi‐MAESTER clones at the clonal and cellular levels. (d) Barcode homoplasy in LARRY clones. The UMAP plot shows that barcode homoplasy results in LARRY clones, such as Clone 17 and Clone 661, consisting of multiple subclones defined by distinct mitochondrial variants. (e) Heatmaps showing refined clonal fates after correction with mitochondrial variants. Each row represents a LARRY clone, and each column corresponds to a specific lineage. (f) Bar chart showing the proportion of clones in uni‐ and multi‐lineages after mitochondrial variant refinement. (g) UMAP plots showing the example mitochondrial variant refined clones with uni‐ (top two) or multi‐lineage (bottom two) characteristics. (h) The bar chart demonstrates that mitochondrial variants significantly increase the proportion of cells committed to specific fates. (i) The RNA velocity field elucidates the fate decisions of major hematopoietic lineages in the LARRY‐MAESTER dataset.

Bulk RNA‐seq of hPSCs (D0) and D18 HEMOs demonstrated substantial overlap in mtDNA variants between stages (Figure 3a), supporting their utility as stable lineage markers. By tracking stem cell subsets marked by unique mtDNA variants, we aim to resolve multi‐progenitor labeling events and refine clonal assignments (Figure 3b). To operationalize this strategy, we designed a targeted mitochondrial variant enrichment assay in LARRY barcoded cells to generate LARRY–MAESTER (Figure 1a), enabling high‐confidence reconstruction of true clonal relationships independent of LARRY technical artifacts.

Informative mitochondrial variants are essential for establishing clonality. However, the absence of a definitive gold standard computational pipeline for mtDNA variant calling and clonal reconstruction presents a significant obstacle to leveraging mtDNA for lineage tracing inference. Presently, two pipelines stand out for mtDNA analysis in single‐cell sequencing data: MQuad and maegatk. Prior to applying these techniques to actual biological datasets, it is essential to conduct a foundational comparison of two available analysis pipelines. This comparative analysis will facilitate an informed decision on the optimal pipeline for analysing real data.

To facilitate the benchmarking work, we generated three Chromium‐MAESTER datasets of HEMOs at D8, D15, and D18. We also included a hematopoietic dataset from the original MAESTER study. To ensure accurate clonality assessment, we initially benchmarked MQuad and maegatk computational pipelines in four HEMO datasets (Figure S6). Subsequent variants identified and clonal assignments derived from processing using both pipelines were used to evaluate the strengths and weaknesses of each pipeline (Figures S7–S24).

In the MQuad pipeline, the number of variants identified in each dataset fell between a similar range of 41 to 44 (Figure S6b and S7), comparing the three organoid datasets. We observed sharp increases in the cumulative distribution of ΔBIC in all three stages (Figure S8), which further justified the rationale behind determining the cutoff based on a knee point and confirmed the informativeness of the identified mitochondrial variants with high sensitivity and specificity. There were significantly fewer variants identified in the hematopoietic dataset (19 variants) (Figures S6b and S9); however, this may be attributed to the size of the dataset being significantly smaller than the other three. We defined 3, 5, 4, and 4 clones by unique combinations of barcodes and reconstructed lineage in D8, D15, and D18 HEMOs, as well as the hematopoietic dataset, respectively (Figures S7 and S9).

For the maegatk pipeline, with the suggested minimum 3 UMI reads parameter, cells with more than 1% VAF for any identified variant were selected and subset for the subsequent clonal assignment step (Figures S10–S14). The variant allele frequency of the selected cells and the variants identified were applied to the R package, clValid [25]. Using the internal validation method, metrics such as connectivity, Dunn index, and silhouette width were applied for validation (Figures S15a, S16a, S17a, and S18a). In all four datasets, the hierarchical clustering method using Euclidean distance metrics was suggested as the best method, this was achieved using the eclust function from the factoextra R package. The ward. D clustering method was applied to accomplish minimal variance between clusters. To aid visualization, the branches of each cluster in the dendrogram were highlighted in a different colour and can be easily interpreted in the simplified dendrogram (Figures S15b, S16b, S17b, and S18b). The number of clonal clusters identified in each of the four datasets is highly varied.

The primary question in evaluating both pipelines remains in their respective capabilities in identifying informative mtDNA variants, which would be useful in downstream analysis of clonal reconstruction for meaningful lineage inference and biological interpretation. When the list of mtDNA variants identified by the two pipelines for each dataset was compared, there was a significantly higher number of variants identified by the maegatk pipeline. However, few overlapping or intersecting variants were identified by both pipelines (Figure S19). This is interesting in understanding that the primary sequencing data used as input for each pipeline is completely identical, and yet the results produced are highly varied.

Heatmaps of scaled VAF values were constructed to visualize the relationship between the variants identified and the clonal structure (Figures S20–S23). In clones assigned using the MQuad pipelines, there was a small but significant number of variants seen to be highly specific to certain clonal clusters (Figures S20a, S21a, S22a, and S23a). This was not observed in the comparatively scattered heatmaps for clones identified using the maegatk pipeline (Figures S20b, S11b, S22b, and S23b). This preliminary result was highly corroborative with the heatmaps produced using the seriation R package, which visually represents the Euclidean distance matrix calculated from the scaled VAF matrix from each pipeline. This suggests that the clones identified by maegatk do not show a clear distinction between clonal clusters using the information supplied by the VAF values of identified variants.

To quantitatively assess the clonal clusters identified using mtDNA informative variants from both pipelines, the Davies‐Bouldin's (DB) index was applied to analyse the clusters identified using the informative variants for each pipeline (Figure S24). A minimal value of the DB index value is indicative of strong clustering, and this was observed in MQuad inferred clonal clusters (Figure S24). The DB index value derived from the maegatk variant inferred clonal clusters were often more than double that in the MQuad clones. The outcome from cluster validation of clonal clusters identified through mtDNA single‐nucleotide variation demonstrated that the variants discovered using the MQuad pipeline are more informative than maegatk. As a result, the MQuad pipeline was used in our study for variant calling and clonal inference.

### 2.5 Orthogonal Mitochondrial Tracing Validates and Refines Clonal Dynamics in HEMOs

To resolve potential technical artifacts in LARRY‐based clonal tracing, we combined LARRY barcoding with mitochondrial variants profiling using MAESTER enrichment to generate LARRY–MAESTER libraries (Figure 1a). From D4 and D8 HEMO LARRY‐MAESTER libraries, MQuad identified 35 and 29 high‐confidence mtDNA variants, respectively. The uneven distribution of mtDNA variants across various cell types suggests the potential to distinguish clonality (Figure S25a). To comprehensively capture cellular correlations at the early stage (D4), we utilized all mtDNA variants for clone assignment, identifying three clones based on unique mitochondrial barcode combinations and lineage reconstruction (Figure S25b). Notably, the mtDNA variants 6321G>A, 6322G>C, 6233A>C, and 6234G>A exhibited high VAF and distinctly marked Clone A0, while another mtDNA variant, 7402C>T, with high VAF, specifically identified Clone A2 (Figure S25b). Interestingly, most cells in Clone_A0 were characterized by mtDNA variant 6321G>A, Clone_A1 predominantly exhibited mtDNA variant 6699G>A, and Clone_A2 was primarily distinguished by mtDNA variant 7402C>A (Figure S25c, d). Neighbourhood analysis revealed strong intra‐clone connectedness, highlighting the ability of mitochondrial variants to accurately delineate robust clonal identities (Figure S25e).

Comparative analysis revealed a striking discordance (52%) between clonal identities defined by LARRY barcodes and those resolved by endogenous mitochondrial variants (Figure S25f). Among multi‐lineage LARRY clones, 208 clones (41.9%) consisted of cells sharing the same mitochondrial variant, providing strong evidence for a pluripotent progenitor origin (Figure 3c). Conversely, 289 multi‐lineage LARRY clones (58.1%) contained more than one mitochondrial clone, indicating they were false positives resulting from LARRY barcode homoplasy (Figure 3c). At the cellular level, only 31.4% of cells could be confidently assigned to pluripotent progenitors based on congruent mitochondrial evidence; the remainder were deemed technical artifacts (Figure 3c). Illustrating this discrepancy, LARRY clones 17 (containing PSC‐Ect, TB‐like, and Mes‐like cells) and 661 (with Mes‐like, EC, and Fibro cells) each comprised three distinct mitochondrial subclones (Figure 3d). This recurrent “one‐to‐many” relationship underscores two critical limitations: pervasive genetic heterogeneity within synthetically defined clones and the high frequency of barcode homoplasy in the LARRY system. The presence of distinct mtDNA variant profiles among cells within a single LARRY clone confirms that such groups are not monoclonal but represent aggregates of biologically independent clones. Given the high conservation of mitochondrial variants between stem cell progenitors and late‐stage (D18) HEMOs in our bulk RNA analysis, we conclude that the observed discordance predominantly reflects intrinsic technical noise of the LARRY system rather than biological variation.

Notably, cells within the 48% concordant clones exhibited more distinct cell type boundaries in the UMAP plot, highlighting the enhanced resolution achieved by integrating endogenous mtDNA markers with exogenous barcoding (Figure S26a). CoSpar analysis further revealed well‐defined cell fates within these concordant clones (Figure 3e). When mitochondrial variants were included, the proportion of uni‐lineage clones increased, while the diversity of multi‐lineage clones decreased, compared with LARRY only clones (Figures 1d,e and 3e,f). For uni‐lineage clones, consistent spatial distributions in UMAP embeddings were observed regardless of whether mitochondrial variants or LARRY barcodes were used. For instance, LARRY Clone 259, belonging to the PSC‐Ect lineage, aligns specifically with mitochondrial Clone_A2, and LARRY Clone 312, representing the Fibro lineage, also maps exclusively to mitochondrial Clone_A2 (Figure 3g).

By contrast, LARRY Clone 515, which spans both PSC‐Ect and TB‐like lineages, is consistently associated with mitochondrial Clone_A1, revealing its bi‐potential nature (Figure 3g, bottom panel). Similarly, LARRY Clone 581, encompassing PSC‐Ect, Fibro, and endoderm lineages, is consistently linked to mitochondrial Clone_A1, highlighting its multi‐potency during HEMO development (Figure 3g, bottom panel). These results suggest that mitochondrial variants not only refine clonal classification but also provide insights into the underlying mechanisms governing lineage commitment and multi‐lineage potential within organoid development.

Interestingly, in the concordant clones, we consistently observed the coupling between the cell fates of Mes‐like and Fibro, as well as Mes‐like and EC cells, further proved the conserved bifurcated differentiation path toward vascular and stromal fates—a hallmark of multipotent mesenchymal progenitor activity (Figure S26b). Statistically, mitochondrial variants significantly increased the proportion of cells with individual cell fate when compared with the LARRY barcoded cell population (Figure 3h).

The mitochondrial‐refined LARRY clones reproduced a similar RNA velocity field to the LARRY‐only clones. Importantly, this refined approach delineated clearer fate‐determination patterns across the principal HEMO lineages, with a particularly notable improvement in the Fibro lineage (Figure 3i). Through likelihood‐based computational screening, we identified putative driver genes governing lineage transitions (Figure S26c). Phase‐plane analysis integrating transcriptional bursting kinetics, velocity vectors, and expression dynamics revealed specific regulatory programs: ZEB2 mediated the terminal differentiation of Mes‐like progenitors (blue) into mature EC (grey) via progressive activation (Figure S26c). This bifurcated architecture indicates early‐stage lineage segregation during hematopoietic specification.

In D8 HEMOs, clonal resolution with mitochondrial variants provides strong support for our conclusion that the system has reached a stable stage of lineage differentiation and structural maturity (Figures S27, S28). The persistence of EC–Fibro coupling—alongside the emerging EMP–EC interaction and clear segregation among other lineages—underscores the continued contribution of mesenchymal and endothelial progenitors in facilitating hematopoietic and stromal crosstalk, even as the system progresses toward compartmentalization (Figure S28).

### 2.6 Spatial mtDNA Clonal Mapping Unveils Niche‐Driven Fate Patterning in Organoids

To validate the ability of the MAESTER‐MQuad algorithm for accurate and reliable clonal tracking in spatial context, we first conducted spatial MAESTER experiments using chondrosarcoma samples. After enriching the mitochondrial transcriptome in the Visium library, MQuad identified 24 informative mitochondrial variants in the Visium‐MAESTER dataset. Utilizing these informative variants, we assigned 1344 chondrosarcoma cells into 16 clones (Figure S29). Notably, we observed that the mitochondrial variant 10310A>G effectively distinguished two major clones: Clone 5 and Clone 11. Clone 11 exhibited a high allelic frequency of 10310A>G, whereas Clone 5 did not show any variation at this position (Figure S29).

When we examined the histoclonal relationship, we observed that Clone 5 was predominantly distributed on the left side of the histological slide, corresponding to the tumor‐adjacent normal area (Figure S30). In contrast, Clone 11 was primarily distributed on the right side of the histological slide, representing the neoplastic tumor area (Figure S30). This suggests that mitochondrial variant 10310A>G is accumulated during the cancer progression process. Our Visium‐MAESTER pipeline is a powerful approach for spatially distinguishing normal clones from neoplastic tumor clones, which provide valuable insights for cell states transition in cancer progression.

Furthermore, we utilized spatialDE [26] to identify gene expressions that significantly depended on spatial coordinates in a non‐linear and non‐parametric manner. Consistent with our MQuad results, spatialDE identified the mitochondrial variant 10310A>G as a significant feature, indicating that the expression of 10310A>G was significantly dependent on its spatial location (Figure S31). Based on these findings, we conclude that the mitochondrial variants identified by MQuad after MAESTER are informative for clonal tracking, and their expression is significantly correlated with their spatial location. Therefore, the clonality established using mitochondrial variants in our Visium‐MAESTER pipeline effectively resolves the spatial architecture of clones.

**FIGURE 4:**
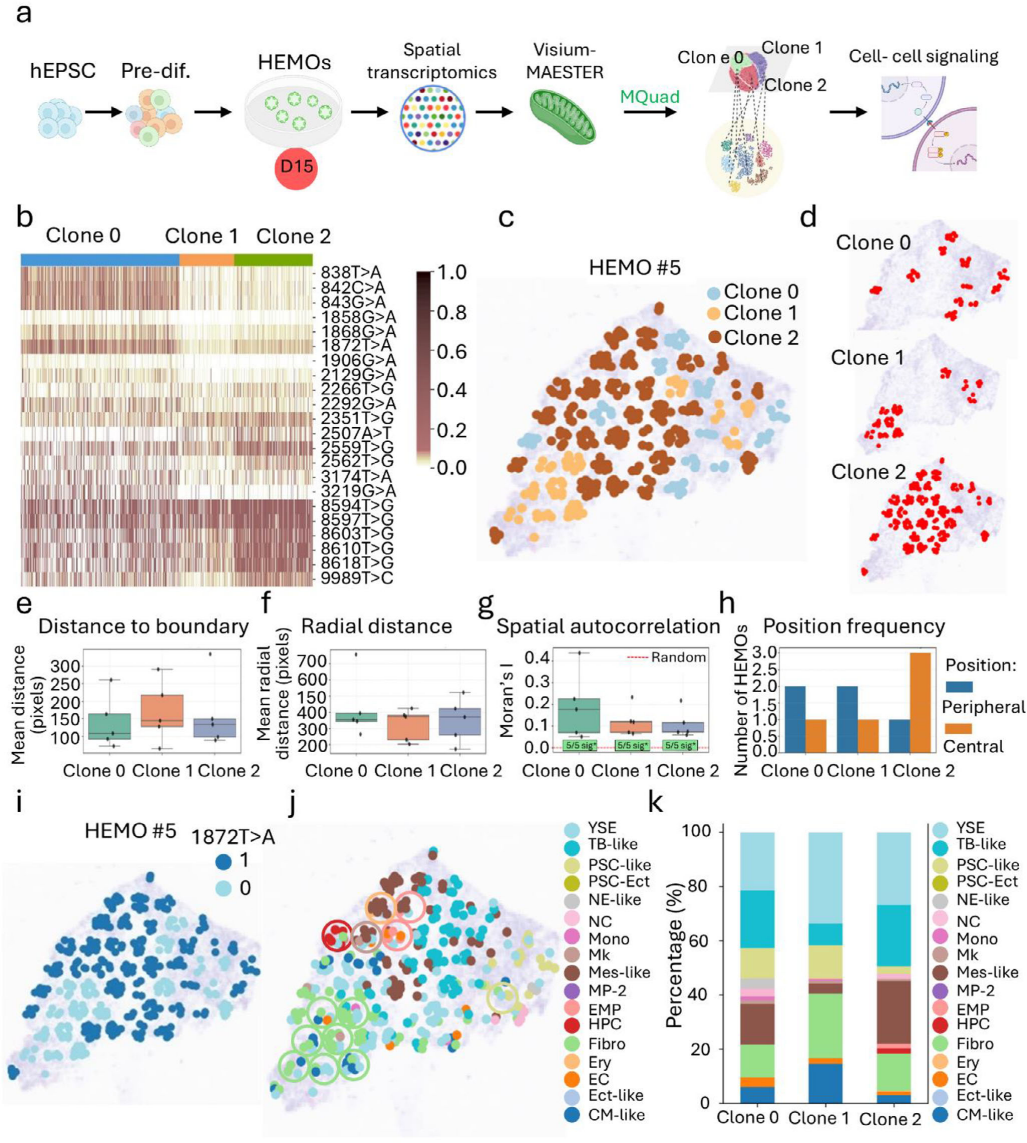
Mitochondrial variants based spatial clonal tracking in D15 HEMOs. (a) Overall study design for mitochondrial variants based spatial lineage tracing. Pre‐differentiated (Pre‐diff.) human expanded pluripotent stem cells (hEPSC) were induced to form human embryonic hematopoietic organoids (HEMOs). HEMOs harvested at Day 15 (D15) were subjected to spatial transcriptomics and mitochondrial variant enrichment to generate Visium‐MAESTER, enabling mitochondrial variants based spatial lineage tracing. (b) Allele frequency heatmap shows the 22 informative mtDNA SNVs detected by MQuad in each clone. Each row is a variant, each column is a barcode. Heatmap color indicates the value of the allele frequency. (c) Spatial architecture of 3 clones identified by mtDNA SNVs from MQuad in Visium‐MAESTER library in HEMO#5. (d) Spatial distribution of individual clones in HEMO #5, highlighting the distinct localization patterns of each clone. (e) Boxplots show the distribution of mean distances to tissue boundary (computed via convex hull) for each clone across five HEMO replicates (*n* = 5 per clone). (f) Boxplots show the distribution of mean radial distances (distance from organoid centroid) for each clone across five HEMO replicates (*n* = 5 per clone). (g) Boxplots show Moran's I values (positive values indicate spatial clustering) for each clone across five HEMO replicates (*n* = 5 per clone). Asterisks indicate significant spatial clustering (*p* < 0.001) in all HEMOs for all clones. The dashed red line (I = 0) represents random spatial distribution. (h) Bar plot shows the number of HEMOs in which each clone was classified as "peripheral" (closest to boundary relative to other clones in same HEMO) or "central" (farthest from boundary). Clone 0 was peripheral in 2/5 HEMOs, Clone 1 in 2/5 HEMOs, and Clone 2 in 1/5 HEMOs; Clone 2 was central in 3/5 HEMOs, Clone 0 in 1/5 HEMOs, and Clone 1 in 1/5 HEMOs. (i) Spatial allelic frequency of mitochondrial SNV 1872T>A, as identified by SpatialDE, aligns strongly with the spatial distribution of Clone 0 and Clone 2 in HEMO #5. This demonstrates the correspondence between mitochondrial variation and clonal spatial patterning. (j) Spatial transcriptomics map of HEMO #5, colored by clusters. The spatial organization of clonal niches is highlighted, with each niche circled and color‐coded to indicate the dominant cell state present within that region. (k) Bar plot shows multiple cell populations in HEMO #5 illustrating the increased formation of HPC and EMP from Clone 1 to Clone 2, as well as increased formation of Mk from Clone 2 to Clone 0 at late stage of hematopoiesis. YSE, yolk sac endoderm; TB‐like, trophoblast‐like cells; PSC‐like, pluripotent stem cells‐like; PSC‐Ect, pluripotent stem cells with ectoderm specification; NE‐like, neural ectoderm‐like cells; NC, neural crest; mono, monocytes; Mk, megakaryocytes; Mes‐like, mesoderm‐like cells; MP‐2, Myeloid progenitor 2; EMP, erythro‐myeloid progenitor; HPC, hematopoietic progenitor cells; Fibro, fibroblasts; Ery, erythroid cells; EC, endothelial cells; Ect‐like, ectoderm‐like cells; CM‐like, cardiac mesoderm‐like cells.

**FIGURE 5:**
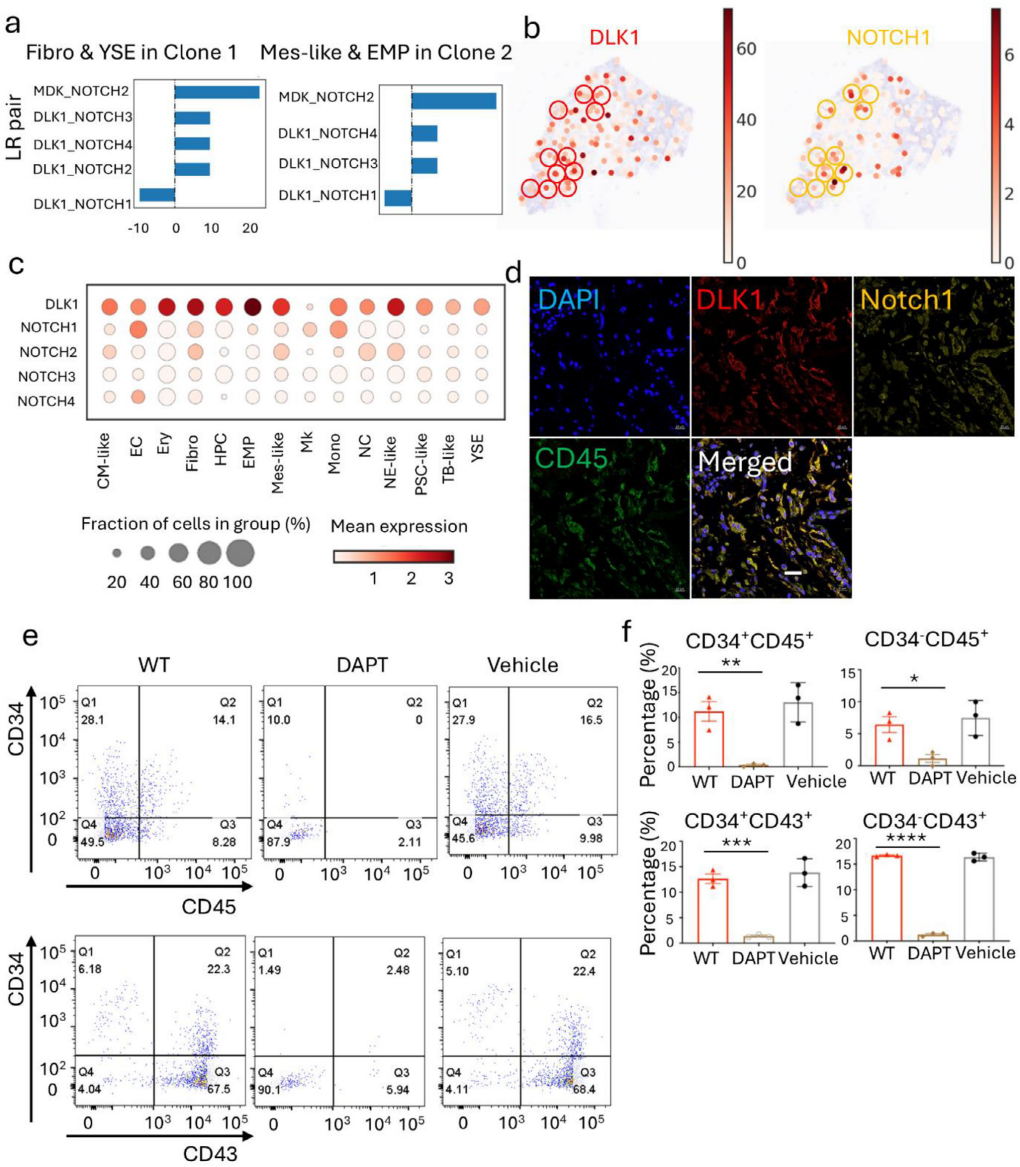
Intercellular signaling drives spatial phenotypic shifts in multicellular neighborhoods of HEMO clones at D15. (a) Spatial cell‐cell interaction analysis conducted by CellPhoneDB within Clone 1 between Fibro and YSE, as well as within Clone 2 between Mes‐like and EMP. The interaction scores (mean value) are shown. Based on curated database annotations, ligand–receptor pairs with a mean score below 0 are classified as inhibitory, while those with a mean score above 0 are classified as activating. The absolute value of the score reflects the interaction strength. (b) Gene expression pattern of *DLK1* and *NOTCH1* within Clone 1 and Clone 2 in HEMO #5 in a spatial slice. *DLK1* highly expressed in YSE within Clone 1 and in EMP within Clone 2. *NOTCH1* highly expressed in Fibro within Clone 1 and in Mes‐like within Clone 2. Circles indicate niches where *DLK1* (red) and *NOTCH1* (yellow) were co‐expressed. (c) Dot plot shows the expression of *DLK1* and *NOTCH1* across various cell types. (d) Immunofluorescence staining of DLK1^+^ hematopoietic cells and Notch1^+^ non‐hematopoietic cells (*n* = 3). Scale bar = 20 µm. (e) Representative flow cytometry dot plots of hematopoietic populations under NOTCH inhibition. Expression of CD34 vs. CD45 (Top)  and CD34 vs. CD43 (Bottom) in WT (untreated control), DAPT‐treated and Vehicle (DMSO) group. Cells were stained with anti‐CD34‐APC and anti‐CD45‐PE or anti‐CD43‐PE antibodies, respectively. Quadrants were set based isotype controls. Percentages of cells in relevant quadrants are indicated. (f) Quantification of key populations defined in (e). (Top) Percentages of CD34^+^CD45^+^ and CD34^+^CD45^−^ cells. (Bottom) Percentages of CD34^+^CD43^+^ and CD34^+^CD43^−^ cells, in Control, Vehicle, and DAPT‐treated groups. Data are presented as mean ± SEM (*n* = 3 independent experiments). Statistical significance was assessed by ordinary one‐way ANOVA followed by Tukey's multiple comparisons test: *p < 0.05, **p < 0.01, ***p < 0.001, ****p < 0.0001.

In light of this validation, we applied spatial transcriptomics in combination with mitochondrial variants to dissect the spatial clonal architecture of hematopoietic and niche cells in D15 HEMOs (Figures 4 and 5). We enriched mitochondrial transcripts from 10x Visium full‐length cDNA and dissected their clonal structure using informative mtDNA variants identified by MQuad (Figure 4a). Across all five HEMOs, we identified a total of 22 informative mtDNA variants (Figure 4b). Among these variants, we found that most of the informative variants were C>T or T>G transitions (Figure S32a). Using vireoSNP, we consistently identified three distinct clones based on these informative variants (Figure 4b). Notably, there is a tendency for the number of variants to increase from Clone 1 to clone 2 and further to Clone 0, suggesting a developmental trajectory among the three clones (Figure 4b). The signature variants 1868G>A, 1906G>A, and 3219G>A are predominantly associated with Clone 0, whereas 2507A>T is primarily observed in Clone 1 and 2 (Figure 4b). By contrast, Clone 1 exhibits a significantly lower number of variants compared to Clones 0 and 2 (Figure 4b). Upon mapping the mtDNA variants to spatial locations, we observed that Clone 0 was primarily located at the edges of organoids, whereas Clone 2 was surrounded by Clone 1 across all five HEMO samples (Figure 4c, d, and Figure S32b).

To rigorously assess spatial patterning, we performed quantitative spatial analysis on all five HEMO replicates (Figure 4e‐h and Table S2). Analysis of distance to the tissue boundary revealed reproducible positional biases among clones (Figure 4e). Clone 0 resided closest to the boundary (140.02 ± 75.87 pixels), indicating a peripheral tendency (Figure 4e). In contrast, Clone 2 showed the strongest central tendency, being classified as the most “central” clone (farthest from the boundary) in 3/5 HEMOs and as “peripheral” in only 1/5 HEMOs. Clone 1 exhibited an intermediate mean boundary distance, typically occupying spatial niches between the peripheral Clone 0 and central Clone 2 domains. Consistent with this spatial organization, radial distance from the centroid measurements confirmed the peripheral localization of Clone 0, which showed the largest mean radial distance (423.74 ± 192.91 pixels) (Figure 4f). Clones 1 and 2 showed progressively smaller radial distances (323.38 ± 99.63 and 350.17 ± 136.91 pixels, respectively), supporting their more central positioning. Furthermore, spatial autocorrelation analysis (Moran's I) demonstrated significant clustering for all three clones across every replicate (Moran's I, *p*  < 0.001), confirming that their distributions are non‐random (Figure 4g).

Using SpatialDE, we found that the expression of 1872T>A was significantly dependent on spatial coordinates. Interestingly, the integrative spatial mapping of both clonality and mitochondrial variants revealed that the high‐expression spatial regions of 1872T>A aligned well with Clone 0 and Clone 2, suggesting the informativeness of mitochondrial variants for dissecting spatial architecture in our organoids (Figure 4i and Figure S30c).

We further aggregated and analysed the cell type composition for each spatial spot based on mtDNA information, revealing that Clones 0 and 2 exhibited greater cellular diversity than Clone 1 across all HEMOs (Figure 4j, k, and Figure S32d, e). Due to the presence of large vacuolated regions in the tissue section of HEMO #2, and the significant reduction in cellular diversity observed in HEMOs #3 and #4, these samples were deemed insufficient to accurately represent the cellular differentiation characteristics of late‐stage HEMOs. Therefore, we selected HEMOs #1 and #5 to study the lineage differentiation and underlying mechanisms of late‐stage HEMOs. In HEMO #5, Clone 1 was characterized by dominance of yolk sac endoderm (YSE) and Fibro, with minimal Mes‐like progenitor activity (Figure 4j, k). This suggests an early bias toward stromal and extraembryonic differentiation, potentially reflecting a niche‐supporting role. Predominantly surrounding Clone 2 of HEMO #5 (Figure 4c, d), Clone 1, with its high Fibro composition, was positioned in a supportive role within the stromal niche. Fibro likely provided structural support or secreted signals regulating neighbouring clones. The presence of PSC‐like cells hinted at retained pluripotency or self‐renewal capacity, while low TB‐like and absent hematopoietic erythroid cells (Ery) and erythro‐myeloid progenitor (EMP) lineages indicated limited trophoblast or blood‐lineage priming of Clone 1 in HEMO #5.

Clone 2 marked a transitional phase, with a resurgence of Mes‐like progenitors alongside a sharp rise in TB‐like cells (Figure 4j, k). Residing in the HEMO core, Clone 2 balanced Mes‐like (23.1%) progenitors with TB‐like (22.7%) and YSE (26.8%) cells, alongside rare hematopoietic progenitor cells (HPC) (1.95%) (Figure 4c, d, j, k). This central niche may act as a developmental crossroads, where mesenchymal progenitors (Mes‐like) transiently reactivate hematopoietic potential (HPC) while seeding trophoblast (TB‐like) and YSE lineages toward peripheral zones. The central position aligned with a “signaling hub” role, coordinating differentiation cues for surrounding clones in HEMO #5.

Significantly localized to the right bottom edge of HEMO #5, Clone 0's dominance of TB‐like (21.1%) and YSE (21.5%) cells, alongside residual Mes‐like (15.0%) and PSC‐like (11.4%) populations, suggests a role in extraembryonic boundary formation. Its peripheral position aligned with TB‐like and YSE lineages, which often demarcate tissue interfaces or nutrient‐exchange zones (Figure 4c, d, j, k). The persistence of PSC‐like cells at the edge hints at a “stemness reservoir” for tissue maintenance or repair at dynamic boundaries.

Similarly, HEMO #1 central clone (Clone 2) consistently occupied dynamic, transitional niches, marked by mixed Mes‐like and lineage‐primed populations (EMP) (Figure S32d, e). This positions it as a differentiation hub directing lineage‐restricted outputs (megakaryocyte/erythroid lineages) rather than retaining multipotent hematopoietic potential. Conversely, peripheral clones (Clone 0) were enriched in stromal (Fibro), extraembryonic (YSE and TB‐like), or niche‐supporting lineages (Figure S32d, e). It lacked PSC‐like cells, suggesting its niche role focused on structural support rather than pluripotency retention. In both HEMO #1 and #5, a shared hierarchy emerges: stromal/extraembryonic‐biased clones (Clone 1) localize to lower/edge zones, while transitional clones (central Clone 2 in both) bridge progenitor states and lineage outputs. Notably, TB‐like and YSE lineages showed spatial polarization, dominating peripheral niches in both HEMOs, likely reflecting conserved roles in boundary formation or nutrient exchange. The persistence of PSC‐like cells at edges and reactivation of HPC or TB‐like potential in cores further suggested a universal tension between stemness maintenance and lineage commitment across spatially partitioned niches. These patterns highlighted a recurring logic: spatial segregation of clones into stemness‐permissive edges and differentiation‐active cores, orchestrated by microenvironmental cues.

### 2.7 NOTCH Signaling Orchestrates Spatial Lineage Transitions via Stromal‐Progenitor Crosstalk in HEMOs

To elucidate the molecular mechanisms underlying this spatial lineage transition during organoid development, we conducted ligand‐receptor (LR) interaction analysis in the aforementioned targeted lineages in both HEMO #1 and #5 (Figure 5, Figures S33–S35). CellPhoneDB revealed a high interaction score for NOTCH ligands and receptors between Fibro and YSE in Clone 1 of HEMO #5 (Figure 5a). The high interaction score was also discovered between Mes‐like and EMP in Clone 2 of HEMO #5 (Figure 5a). Intrigued by the critical role of NOTCH signaling in developmental hematopoiesis [27, 28, 29], we quantified both the activation and inhibition of NOTCH signaling based on the combination of ligand‐receptor interactions (Figure 5b, c). In HEMO #5, YSE expressed Delta‐Like Non‐Canonical Notch Ligand 1 (DLK1), while Fibro expressed Notch Receptor 1 (NOTCH1) in the same spatial area within Clone 1 (Figure 5b, c). Similarly, EMP expressed DLK1, while Mes‐like expressed NOTCH1 in the same spatial region of Clone 2 (Figure 5b, c). DLK1 regulates the hematopoietic progenitor pool by suppressing proliferation and differentiation [30, 31]. Immunofluorescence staining confirmed the spatial organization of this niche (Figure 5d), highlighting the pivotal role of NOTCH signaling in both clonal architecture and niche regulation within human embryonic organoid models.

To move beyond correlation and establish a causal role for the observed NOTCH signaling networks, we functionally perturbed NOTCH cleavage using the gamma‐secretase inhibitor DAPT. HEMOs were treated with DAPT and analyzed at a key developmental timepoint (Day 12), alongside untreated controls (Figure 5e, f). Flow cytometric analysis revealed that pharmacological inhibition of NOTCH signaling markedly altered the composition of developing hematopoietic populations. Specifically, DAPT treatment induced distinct changes in the abundance of CD34^+^CD45^−^, CD34^+^CD45^+^, CD34^+^CD43^−^, and CD34^−^CD43^+^ cell populations. In control (WT) samples, gating on CD34 versus CD45 expression identified prominent CD34^+^CD45^+^ and CD34^−^CD45^+^ subsets, representing 14.1% and 8.28% of total cells, respectively (Figure 5e). In stark contrast, DAPT treatment drastically reduced these populations to 0% and 2.11%, respectively. A parallel analysis of CD34 versus CD43 expression yielded consistent results. In WT controls, CD34^+^CD43^+^, and CD34^−^CD43^+^ subsets constituted 22.3% and 67.5% of cells, respectively. Following DAPT inhibition, these populations were similarly diminished, dropping to 2.48% and 5.94%. In both analyses, vehicle‐treated controls exhibited profiles similar to the untreated WT group. We also assessed the cell viability. Comparison between DAPT‐treated, vehicle control, and untreated WT groups revealed no significant difference in overall cell viability (viability > 80% across all groups; Figure S33d). This confirms that the observed shifts in population composition are not due to nonspecific cytotoxic effects of the treatment. These results demonstrate that NOTCH signaling is essential for the establishment of the hemogenic lineage and its subsequent progression into committed blood cells during the HEMO differentiation process.

## 3 Discussion

By integrating endogenous mitochondrial lineage tracing with single‐cell and spatial transcriptomics, we establish a multimodal framework that reconstructs high‐resolution clonal dynamics and uncovers principles of tissue self‐organization during human hematopoietic organoid (HEMO) development. Our integrated profiling delineates a hierarchical roadmap of cell fate decisions within HEMOs. The early coupling of Mes‐like progenitors to both endothelial and stromal fates mirrors the in vivo patterning of the lateral plate mesoderm, as defined in comprehensive maps of human mesoderm development [24]. Our immunofluorescence and live‐imaging data capture a differentiation trajectory in which PDGFRα^+^ mesenchymal progenitors give rise first to α‐SMA^+^ fibroblasts and subsequently to CD31^+^ endothelial cells (Figure 1i and Movie S1). This temporal sequence provides experimental evidence that aligns with the lineage couplings and transcriptional dynamics predicted by our RNA velocity model (Figure 1 g). This temporal sequence of lineage specification underscores that HEMOs recapitulate the very early stages of hematopoietic niche formation, where the specification of vascular and stromal components is a prerequisite for hematopoiesis. Furthermore, the transcriptional identity and differentiation potential of our D4 progenitors align them more closely with mesodermal populations found in the early yolk sac and aorta‐gonad‐mesonephros (AGM) region, which are responsible for generating the first wave of erythro‐myeloid progenitors and establishing the hemogenic endothelium, rather than with mature, transplantable HSCs that emerge later in the fetal liver [32]. The spatial zonation of clones we observed, with distinct progenitor states occupying specific microniches, further echoes the anatomical segregation of developmental hematopoiesis across different embryonic compartments.

Our study establishes mtDNA variants as endogenous genetic recorders that enable high‐resolution reconstruction of spatiotemporal clonal dynamics during HEMO development. By integrating mtDNA‐based lineage tracing with single‐cell and spatial transcriptomic profiling, we uncover fundamental principles of cell fate decisions and tissue self‐organization that reflect conserved developmental processes reminiscent of early human embryogenesis. These findings significantly advance our understanding of in vitro hematopoiesis and provide a conceptual and technical framework for investigating lineage segregation and clonal architecture in complex cellular ecosystems.

A major insight arising from our analysis is the striking spatial zonation of clonal units within HEMOs. We found that clones are not randomly distributed but occupy distinct microniches: peripheral regions were enriched in pluripotent and extraembryonic (TB‐like, lineages, whereas central areas harbored more mesenchymal and hematopoietic progenitors. This structural organization—which we term “stemness‐permissive edges and differentiation‐active cores”—parallels spatial patterning phenomena observed in vertebrate embryos and mirrors mechanisms observed in early embryogenesis, suggesting the influence of conserved biophysical or biochemical gradients on cell fate specification [33]. The clear correlation between clonal identity and spatial context underscores that organoid development is not solely cell‐intrinsic but is profoundly shaped by positional cues, potentially mediated through morphogen gradients, cell‐cell contact, and metabolic microenvironments.

Notably, ligand‐receptor analysis provided mechanistic insight into this spatial patterning by revealing enriched NOTCH signaling between stromal (Fibro, YSE, and Mes‐like) and progenitor (EMP) cells. This finding resonates with the essential role of NOTCH signaling during in vivo embryonic hematopoiesis, particularly in the AGM region generation of hematopoietic stem cells (Espinosa & Bigas, 2011), modelled in this study. Our comparative analysis indicates that HEMOs recapitulate many features of yolk sac and early AGM‐stage hematopoiesis, including the emergence of EMP and endothelial commitment. That said, the absence of robust lymphoid differentiation and inter‐organoid variability highlights remaining limitations. Nevertheless, the faithful reconstruction of NOTCH‐mediated signaling and the spatial organization of clonal structure reinforce the physiological relevance of this model for studying human hematopoietic development.

An important methodological contribution of our work is the demonstration that mtDNA variants can significantly improve confidence in lineage tracing. Recent studies have pioneered the use of mtDNA variants to track clonal dynamics in contexts such as aging hematopoiesis and cancer evolution [34, 35]. However, our work is the first to apply and validate this approach for high‐resolution lineage tracing in a developing human organoid model. We found that nearly 60% of putative multi‐lineage clones identified by the LARRY barcoding system were actually polyclonal aggregates—a result of barcode homoplasy rather than true multipotency. The use of mtDNA variants enabled us to distinguish such technical artifacts from legitimate multipotent progenitors, including clones capable of producing both ectodermal and trophoblastic lineages, a fate combination seldom reported in conventional differentiation systems. This orthogonal approach greatly enhances the accuracy of lineage reconstruction in densely populated organoid systems.

During the preparation of this manuscript, a related method called SUMMIT was reported for spatially‐resolved mitochondrial lineage tracing in intact human tissues [36]. Both studies employ targeted enrichment of mitochondrial transcripts from spatial cDNA libraries and computationally identify informative mtDNA variants to reconstruct clonal architecture. This methodological convergence underscores the robustness and utility of mtDNA variants as endogenous, spatially‐compatible lineage recorders. While MAESTER‐MQuad has proven effective, its analytical strategy differs fundamentally from that of SUMMIT. Specifically, SUMMIT applies a binomial regression model to detect cell type‐enriched mitochondrial variants, while MQuad relies on a binomial mixture model to select clone‐informative variants. Consequently, as an unsupervised method, MQuad is more unbiased and capable of detecting clonal variants shared across multiple cell types. SUMMIT, as a supervised approach, offers greater robustness and sensitivity—though typically only when the variants are cell type‐specific.

While our study provides functional validation for key lineage trajectories and NOTCH signaling through perturbation and imaging, the multipotency of specific clonal units, as defined by mitochondrial barcodes, awaits final confirmation through single‐cell‐derived differentiation assays or transplantation. Looking forward, the multimodal framework developed here offers multiple promising avenues. Having established the necessity of NOTCH signaling, future work can now dissect its sufficiency and precise cellular source within the niche through targeted genetic editing. Applying this platform to disease‐specific iPSCs could elucidate clonal dynamics in genetic blood disorders or leukemias.

In conclusion, by integrating computational lineage tracing with targeted experimental validations, our work establishes mtDNA variants as powerful natural barcodes for reconstructing lineage relationships and spatial clonal dynamics in human organoids. Beyond providing technical corrections to synthetic barcoding systems, we demonstrate that clonal architecture is intimately linked to spatial niche identity, unveiling patterning principles that underlie early blood development. This integrated approach offers a new foundation for studying human development, disease modeling, and regenerative medicine with unprecedented spatial and clonal resolution.

## 4 Methods

### 4.1 HEMO Differentiation

HEMO differentiation was done based on the previously established protocol [37]. In brief, we formed embryoid bodies (EBs) from hPSCs and differentiated to mesoderm and hematoendothelial lineage by morphogens and cytokines.

#### 4.1.1 hPSCs Line

Human M1‐hPSCs were gifted by Pentao Liu and maintained in the media described before [38].

#### 4.1.2 hPSCs Maintenance and Pre‐Differentiation

The hPSCs were cultured and maintained in PSC medium, with medium changes occurring every other day. The composition of the medium was previously reported [38]. Prior to the formation of EBs, cells were pre‐differentiated in KSR medium (DMEM/F12 + 10% KSR) (Thermo Scientific, catalog no. 11320033; Thermo Scientific, catalog no. 10828028) for a period of three days.

#### 4.1.3 EB Formation in Hanging Drop

To begin the process, the KSR medium was removed, and the cells were washed with PBS (Thermo Scientific, catalog no. 10010023). The cells were then digested with 500 mL of 0.05% Trypsin (Thermo Scientific, catalog no. 25300054) at 37°C for three minutes, but no longer than seven minutes. The Trypsin was removed, and 2 mL of KSR medium was added to harvest the cells. Careful handling of the cells was necessary, and harsh pipetting was to be avoided. The cells were then centrifuged at 300 g for three minutes, and the supernatant was carefully removed. To resuspend the cells, 1 mL of KSR medium and 1 µL of Y27632 (10 ug/ml) (Tocris, catalog no. 1254/10) were added. For each 25 µL hanging drop on the cap of a 10 cm petri dish, 4,000 cells were added. Thirty to forty drops were made for each cap, and the dish was filled with PBS to keep it moist. The cap was then gently and slightly inverted to cover the dish, and the dishes were labeled and kept at 37°C for three days.

#### 4.1.4 EB Collection and Differentiation

After the formation of EBs, all EBs were collected and washed with PBS. The EBs were then centrifuged at 100 g for one minute, and the supernatant was carefully removed. 1 mL of medium A (STEMdiff Hematopoietic Kit, catalog no. 05310) was added, and the EBs were transferred to a non‐adherent 24‐well plate. The date was labeled as Day 0, and the EBs were cultured with 1 mL of STEMdiff medium A for three days. On Day 2, the medium was half‐changed, and the EBs were subsequently cultured with 1 mL of STEMdiff medium B for the following days. This process involved the maintenance of hPSCs, pre‐differentiation, EB formation, collection, and differentiation.

#### 4.1.5 LARRY Barcoding

We performed LARRY barcoding on two independent batches of pre‐differentiated hPSCs to generate D4 and D8 HEMOs. hPSCs were passaged one day prior to the experiment using TrypLE (Gibco, Cat. #12605036) and seeded in a 6‐well plate at a density of 8 × 10^5^ cells per well in PSC medium. The following day, the medium was replaced with DFK medium (DMEM/F12 supplemented with 20% serum replacement, 1% penicillin‐streptomycin, and 1× GlutaMAX) to initiate pre‐differentiation. After three days, the cells were digested with TrypLE. Feeder cells were removed by filtering the suspension through a 0.4 µm cell strainer, and the cells were counted.

To generate EBs and barcode the cells, the digested cells were resuspended at a concentration of 1 × 10^5^ cells/mL in DFK medium supplemented with 10 µm Y27632 (MCE, Cat. #HY‐10071). LARRY lentivirus was added at a multiplicity of infection (MOI) of 0.1, and the mixture was thoroughly vortexed. We then pipetted 30 µL droplets of the cell‐virus mixture onto the lid of a 10 cm dish, the bottom of which contained 10 mL of PBS to maintain humidity. The day of droplet generation was designated as Day 0 (D0). After the droplets had settled and formed EBs by the following day (Day 1), the lid was carefully inverted to initiate hanging drop culture. Three days after inversion (Day 4), the barcoded EBs were harvested using a 1 mL pipette and resuspended in Medium A. The harvested EBs were sorted for the GFP‐positive population. These sorted cells were subsequently processed for scRNA‐seq using the 10X Chromium platform and for next‐generation sequencing (NGS) to generate the D4 LARRY dataset.

For the D8 LARRY dataset, EBs collected at D4 from an independent batch were transferred to a low‐attachment 24‐well plate (SPL, Cat. #SPL‐11245). After two days, half of the medium was replaced with fresh Medium A. On the following day, the medium was switched to Medium B (Stemcell, Cat. #05310). On day 8 (D8), the barcoded HEMOs were harvested, and the GFP‐positive population was sorted for subsequent processing via 10X Chromium scRNA‐seq and NGS.

### 4.2 Chondrosarcoma Sample

The current study was approved by the Institutional Review Board of the University of Hong Kong/Hospital Authority Hong Kong West Cluster (IRB reference number: UW 16‐2036). The patient sample was obtained from Queen Mary Hospital, Hong Kong, following informed consent from the patient. The fresh specimen was collected during surgical resection and immediately transported on ice to maintain tissue integrity. Diagnosis and grading were performed by a multidisciplinary team of pathologists and orthopaedic surgeons. The specimen was stored at −80°C prior to processing.

### 4.3 10x Genomics Chromium scRNA‐seq

To prepare single‐cell suspensions, organoids were harvested at D8, D15, D18, D25, and D32 since EB formation in hanging drop. After washing with PBS, organoids were mechanically chopped with scissors for 20–30 times, followed by digestion with 500 µL Accumax at 37 °C for 10–15 min. Digestion was terminated by 500 µL PBS containing 2% FBS. Cells were filtered with a 40 µm cell filter and centrifuged at 500 × g for 5 min. The collected cells were resuspended in FACS sorting buffer (1 × PBS with 2% FBS), and their concentration was adjusted to around 3k cells per µL by counting with a hemocytometer.

Subsequently, cells were stained with DAPI (BD Biosciences, catalog no. 564907) in 1:100 for 5 min at 4 °C and were sorted by BD Influx flow cytometry in CPOS at HKUMed. Cells were gated to exclude dead cells and doublets and collected in a chilled PBS with 0.04% BSA for scRNA‐seq library construction. Cell concentration was adjusted to around 500–1000 cells per µL.

10x Genomics 3′ Single Cell Gene Expression v3 reagents were used for scRNA‐seq library preparation. Single‐cell encapsulation and reverse transcription were performed at the Centre for PanorOmic Sciences, the University of Hong Kong. Briefly, 8–16k live single cells of size 30 µm or smaller and of good viability were encapsulated in gel bead‐in emulsions. Captured mRNAs were transcribed into cDNAs and amplified to generate whole‐transcriptome amplifications (WTAs). Libraries were constructed by fragmentation, adapter ligation, and a sample index PCR. Libraries were sequenced using Illumina Novaseq 6000 for Pair‐End 151 bp sequencing.

### 4.4 10x Genomics Visium Library Construction and Sequencing

The Visium Spatial Tissue Optimization Slide & Reagent kit (10x Genomics, catalog no. PN‐1000193) was used to optimize permeabilization conditions for the tissue sections. The Visium Spatial Gene Expression Slide & Reagent kit (10X Genomics, catalog no. PN‐1000184) was used to generate spatially barcoded cDNA from every 10‐µm sections of D15 HEMO samples. The Library construction kit (10 x Genomics, catalog no. PN‐1000190) was used for library construction. Frozen HEMO sample preparation, RNA integrity detection, tissue optimization, staining and imaging, cDNA synthesis and second strand synthesis, cDNA amplification and library construction were carried out according to Chao et al. [37] Libraries were sequenced on Illumina sequencing instruments using TruSeq Read 1 and TruSeq Read 2 primers.

### 4.5 Mitochondrial Alteration Enrichment for Chromium‐MAESTER and Visium‐MAESTER

MAESTER was performed according to Miller et al. [39]. Briefly, WTAs yield in high‐throughput 10x Genomics scRNA‐seq 3’ and Visium platforms served as initial templates for the enrichment of 15 mitochondrial transcripts by two rounds of PCR. Sequences of the amplified mitochondrial transcripts were obtained by standard next‐generation sequencing.

In PCR1, twelve primer mixes were created using the designed primers tiled across the entire mitochondrial transcriptome (Table 1). A barcoded sample indexing i5 primer was included in each mix, in which 20 ng WTAs were included. PCR was performed using the following conditions: denaturation at 95°C for 3 min, followed by 6 cycles of 98°Cfor 20 s, 65°Cfor 15 s, and 72°Cfor 3 min, ending with a final extension at 72°Cfor 5 min. Following amplification, the PCR products were pooled in a certain ratio and purified with 1x AMPure XP beads (Beckman Coulter A63881).

**TABLE 1:** Primers used for mitochondrial variants enrichment.

PCR Mix	Primer name	Complete sequence (5’to 3’)
1	PvG1218_MT‐ND1_4009	CACCCGAGAATTCCAAACACCCTCACCACTACAATCT
1	PvG1223_MT‐ND2_5363	CACCCGAGAATTCCACTCCACCTCAATCACACTACTCC
1	PvG1230_MT‐CO1_7184	CACCCGAGAATTCCAACAACACTTTCTCGGCCTATCC
1	PvG1234_MT‐ATP8_8367	CACCCGAGAATTCCATGCCCCAACTAAATACTACCG
1	PvG1241_MT‐CO3_9756	CACCCGAGAATTCCATCTCCCTTCACCATTTCCGAC
1	PvG1243_MT‐ND3_10264	CACCCGAGAATTCCATTGCCCTCCTTTTACCCCTAC
1	PvG1244_MT‐ND4L_10496	CACCCGAGAATTCCAACTAGCATTTACCATCTCACTTCT
1	PvG1251_MT‐ND4_11900	CACCCGAGAATTCCAGTGCTAGTAACCACGTTCTCCT
1	PvG1259_MT‐ND5_13926	CACCCGAGAATTCCATAGCATCACACACCGCACAA
1	PvG1260_MT‐ND6_14263	CACCCGAGAATTCCAGGATCCTATTGGTGCGGGG
1	PvG1268_MT‐CYB_15643	CACCCGAGAATTCCACATCCTAGCAATAATCCCCATCCT
2	PvG1217_MT‐ND1_3777	CACCCGAGAATTCCATGGCTCCTTTAACCTCTCCAC
2	PvG1222_MT‐ND2_5145	CACCCGAGAATTCCAACGACCCTACTACTATCTCGCA
2	PvG1229_MT‐CO1_6957	CACCCGAGAATTCCAGGCCTGACTGGCATTGTATT
2	PvG1232_MT‐CO2_7852	CACCCGAGAATTCCAGGTCAACGATCCCTCCCTTAC
2	PvG1236_MT‐ATP6_8766	CACCCGAGAATTCCACACAACTAACCTCCTCGGACT
2	PvG1240_MT‐CO3_9535	CACCCGAGAATTCCACCCAATTAGGAGGGCACTGG
2	PvG1242_MT‐ND3_10127	CACCCGAGAATTCCAACTACCACAACTCAACGGCTAC
2	PvG1250_MT‐ND4_11684	CACCCGAGAATTCCATTCACCGGCGCAGTCATT
2	PvG1258_MT‐ND5_13758	CACCCGAGAATTCCACGCATCCCCCTTCCAAACA
2	PvG1261_MT‐ND6_14492	CACCCGAGAATTCCAGGGGAATGATGGTTGTCTTTGG
2	PvG1267_MT‐CYB_15432	CACCCGAGAATTCCACCCTCGGCTTACTTCTCTTCC
3	PvG1216_MT‐ND1_3537	CACCCGAGAATTCCAAGCTCTCACCATCGCTCTTC
3	PvG1221_MT‐ND2_4923	CACCCGAGAATTCCAAGCCTTCTCCTCACTCTCTCAA
3	PvG1228_MT‐CO1_6742	CACCCGAGAATTCCATTGGCTTCCTAGGGTTTATCGTG
3	PvG1231_MT‐CO2_7609	CACCCGAGAATTCCATCTACAAGACGCTACTTCCCC
3	PvG1235_MT‐ATP6_8541	CACCCGAGAATTCCAGTTCGCTTCATTCATTGCCCC
3	PvG1239_MT‐CO3_9316	CACCCGAGAATTCCATCCACTCCATAACGCTCCTC
3	PvG1249_MT‐ND4_11491	CACCCGAGAATTCCAACGCCTCACACTCATTCTCAA
3	PvG1257_MT‐ND5_13515	CACCCGAGAATTCCACCACATCATCGAAACCGCAAA
3	PvG1262_MT‐ND6_14664	CACCCGAGAATTCCAGCTTTGTTTCTGTTGAGTGTGG
3	PvG1266_MT‐CYB_15260	CACCCGAGAATTCCAAGTCCCACCCTCACACGAT
4	PvG1204_MT‐RNR1_656	CACCCGAGAATTCCATGGTCCTAGCCTTTCTATTAGCTC
4	PvG1215_MT‐ND1_3398	CACCCGAGAATTCCATACAACTACGCAAAGGCCCC
4	PvG1220_MT‐ND2_4711	CACCCGAGAATTCCACCGGACAATGAACCATAACCAA
4	PvG1227_MT‐CO1_6547	CACCCGAGAATTCCATCAACACCACCTTCTTCGACC
4	PvG1238_MT‐CO3_9210	CACCCGAGAATTCCAACCCACCAATCACATGCCTATC
4	PvG1248_MT‐ND4_11410	CACCCGAGAATTCCATAAAGCCCATGTCGAAGCCC
4	PvG1256_MT‐ND5_13288	CACCCGAGAATTCCAGGCATCAACCAACCACACCT
4	PvG1265_MT‐CYB_15088	CACCCGAGAATTCCACATCGGCATTATCCTCCTGCT
5	PvG1219_MT‐ND2_4483	CACCCGAGAATTCCACCCAACCCGTCATCTACTCTAC
5	PvG1226_MT‐CO1_6324	CACCCGAGAATTCCAGCCTCCGTAGACCTAACCATC
5	PvG1247_MT‐ND4_11223	CACCCGAGAATTCCATAGGCTCCCTTCCCCTACTC
5	PvG1255_MT‐ND5_13069	CACCCGAGAATTCCAGCCCTACTCCACTCAAGCAC
5	PvG1264_MT‐CYB_14937	CACCCGAGAATTCCACATCAATCGCCCACATCACTC
6	PvG1212_MT‐RNR2_2524	CACCCGAGAATTCCAACCAGTATTAGAGGCACCGC
6	PvG1225_MT‐CO1_6124	CACCCGAGAATTCCATAATCGGAGGCTTTGGCAACT
6	PvG1246_MT‐ND4_10994	CACCCGAGAATTCCAGCAAGCCAACGCCACTTATC
6	PvG1254_MT‐ND5_12831	CACCCGAGAATTCCACACAGCAGCCATTCAAGCAA
6	PvG1263_MT‐CYB_14789	CACCCGAGAATTCCAAACCACTCATTCATCGACCTCC
7	PvG1210_MT‐RNR2_2110	CACCCGAGAATTCCAACAGCTCTTTGGACACTAGGAA
7	PvG1224_MT‐CO1_5910	CACCCGAGAATTCCAGCCGACCGTTGACTATTCTCT
7	PvG1245_MT‐ND4_10761	CACCCGAGAATTCCATGCTAAAACTAATCGTCCCAACAA
7	PvG1253_MT‐ND5_12601	CACCCGAGAATTCCATTCATCCCTGTAGCATTGTTCGT
8	PvG1208_MT‐RNR2_1679	CACCCGAGAATTCCATAGCCCCAAACCCACTCCAC
8	PvG1252_MT‐ND5_12360	CACCCGAGAATTCCACACCCTAACCCTGACTTCCC
9	PvG1207_MT‐RNR1_1347	CACCCGAGAATTCCAGGTGGCAAGAAATGGGCTACA
9	PvG1214_MT‐RNR2_2985	CACCCGAGAATTCCACCTCGATGTTGGATCAGGAC
9	PvG1237_MT‐ATP6_8992	CACCCGAGAATTCCACTGGCCGTACGCCTAACC
10	PvG1206_MT‐RNR1_1127	CACCCGAGAATTCCAACTGCTCGCCAGAACACTAC
10	PvG1213_MT‐RNR2_2757	CACCCGAGAATTCCAAGTACCTAACAAACCCACAGGTC
11	PvG1205_MT‐RNR1_899	CACCCGAGAATTCCAGCGGTCACACGATTAACCCA
11	PvG1211_MT‐RNR2_2323	CACCCGAGAATTCCAATTCTCCTCCGCATAAGCCTG
12	PvG1209_MT‐RNR2_1895	CACCCGAGAATTCCACTAAGACCCCCGAAACCAGA

The purified products were used as the template for PCR2 in which the Illumina adapters (P5, P7), dual index barcodes to identify the sample (i5, i7), and sequencing primer binding sites to the fragments were added. The programme of PCR2 was an initial denaturation at 95°Cfor 3 min, then 6 cycles of 98°Cfor 20 s, 60°Cfor 30 s, and 72°Cfor 3 min, and then a final extension at 72°Cfor 5 min. After PCR2, the DNA was purified with 0.8x AMPure XP beads. The library concentration of three Chromium‐ and Visium‐MAESTER samples were 50, 59, 71 and 21 ng/mL respectively. Resulting MAESTER and Visium‐MAESTER libraries were sequenced on the Illumina NovaSeq SP PE150 kit with run cycle 28, 8, 8, 256 for Read 1, i7, i5 and Read 2 respectively. The sequences of Nove seq primers are listed in Table 2.

**TABLE 2:** Primers used for Illumina NovaSeq.

Primer name	Complete sequence (5’to 3’)
D8‐i7‐BC	CAAGCAGAAGACGGCATACGAGATGGTCCAGAGTGACTGGAGTTCCTTGGCACCCGAGAATTCCA
D15‐i7‐BC	CAAGCAGAAGACGGCATACGAGATGCACATCTGTGACTGGAGTTCCTTGGCACCCGAGAATTCCA
D18‐i7‐BC	CAAGCAGAAGACGGCATACGAGATTTCGCTGAGTGACTGGAGTTCCTTGGCACCCGAGAATTCCA
Visium‐i7‐BC	CAAGCAGAAGACGGCATACGAGATAGCAATTCGTGACTGGAGTTCCTTGGCACCCGAGAATTCCA
D8‐i5‐BC	AATGATACGGCGACCACCGAGATCTACACCGGTTCTTTCTTTCCCTACACGACGCTC
D15‐i5‐BC	AATGATACGGCGACCACCGAGATCTACACAACCTCTTTCTTTCCCTACACGACGCTC
D18‐i5‐BC	AATGATACGGCGACCACCGAGATCTACACCGCATATTTCTTTCCCTACACGACGCTC
Visium‐i5‐BC	AATGATACGGCGACCACCGAGATCTACACCTGCTCCTTCTTTCCCTACACGACGCTC

### 4.6 Immunofluorescence Staining for Single‐Cell Suspensions

PDGFRα^+^ cells were isolated from wild‐type (WT) organoids via flow cytometry at Day 2 of differentiation. The sorted cells were cultured in StemDiff media for +2 days (D4) or +6 days (D8), then processed for immunofluorescence staining, respectively. Cells were pelleted by centrifugation at 500 g for 5 min and subsequently deposited onto glass slides using a cytocentrifuge (800 g, 3 min). The slides were air‐dried overnight prior to staining.

The dried cell spots were fixed with 4% paraformaldehyde (PFA; Beyotime, P0099) for 10 min at room temperature (RT), followed by a PBS wash. After outlining the sample area with a hydrophobic barrier pen, cells were permeabilized and blocked simultaneously by incubation with a solution of 0.1% Triton X‐100 and 1% bovine serum albumin (BSA; Sigma‐Aldrich, 93443) in PBS for 30 min at RT.

Following permeabilization, non‐specific binding sites were blocked with a solution of 10% fetal bovine serum (FBS) in PBS for 1–2 h at RT. Cells were incubated overnight at 4°C with primary antibodies diluted in blocking buffer (10% FBS in PBS). The following primary antibodies were used: mouse anti‐human CD31 (1:100; Abcam, ab9498) and rabbit anti‐human α‐SMA (1:100; Abcam, ab5694). The next day, slides were washed three times with PBS and incubated with species‐appropriate secondary antibodies (Anti‐rabbit and Anti‐mouse IgG) conjugated to fluorophores, diluted at 1:5000 in blocking buffer, for 30 min at RT in the dark. After incubation, slides were washed three times with PBS. Nuclei were counterstained with DAPI (BD Biosciences, 564907) for 3 min, followed by a final PBS wash. Slides were mounted with an anti‐fade mounting medium and sealed with clear nail polish.

Confocal images were acquired using a Carl Zeiss LSM900 microscope (Centre for PanorOmic Sciences, Li Ka Shing Faculty of Medicine, The University of Hong Kong). Image processing and analysis were performed using ImageJ software.

PDGFRα^+^ cells were isolated from organoids via flow cytometry at Day 2 of differentiation. The sorted cells were also cultured in StemDiff media and proceeded with time‐lapse imaging overnight with Nikon Ti2‐E microscopy.

### 4.7 Immunofluorescence Staining Histological Slides

D15 HEMO samples were paraffin‐embedded. The subsequent fixation, sectioning, dehydration, permeabilization, and antibody incubation were performed according to Chao et al., 2023 [37]. Following the washing step with PBS twice, the slides were maintained in a wet state. Subsequently, images were captured using a Nikon Ti2E Fluorescent microscope and merged using ImageJ software. Antibodies for hematopoietic cells were anti‐CD45 antibody (Invitrogen catalog no. MA5‐17687) and Goat Anti‐Rabbit IgG H&L (Abcam catalog no. ab150157). Antibodies for DLK1 were anti‐DLK‐1 antibody (Abcam catalog no. ab89908) and Goat Anti‐Mouse IgG H&L (Abcam catalog no. ab175473). Antibodies for Notch1 were anti‐Notch1 antibody (Abcam catalog no. ab245686) and Goat Anti‐Rabbit IgG H&L (Abcam catalog no. ab150079).

### 4.8 Inhibition of NOTCH Signaling During HEMO Differentiation

The human EPSC cell line was maintained in culture medium. To initiate differentiation, EBs were formed in DMEM/F12 medium (Thermo Fisher Scientific, 21331020) supplemented with 10 µm Y‐27632 (MCE, HY‐10583). EBs were cultured for 3 days in StemDiffA medium (STEMCELL Technologies, 05310) to induce mesoderm patterning, with a half‐medium change on day 1. The medium was then switched to StemDiffB (STEMCELL Technologies, 05310) to direct hemato‐endothelial differentiation for an additional 7 days.

For the NOTCH inhibition group, DAPT (MCE, HY‐13027) was added to the culture medium at a final concentration of 50 µM starting from day 0. The untreated control (WT) and Vehicle groups (DMSO) were cultured in parallel without DAPT. Following differentiation, organoids from the WT, DAPT‐treated, and Vehicle groups were harvested and dissociated into single‐cell suspensions. Cells were washed in PBS and resuspended in homemade FACS buffer (PBS containing 2% FBS). To minimize non‐specific binding, cells were incubated with an Fc receptor blocking solution (BioLegend, 422302; 5 µL per sample) for 10 min at room temperature. Cells were then stained with the following antibody cocktail for 30 min at 4°C in the dark: CD31‐PE‐Cy7/CD45‐APC, CD34‐APC/CD45‐PE, and CD34‐APC/CD43‐PE. After staining, cells were washed twice with ice‐cold FACS buffer and resuspended in buffer containing 1 µg/mL DAPI for live/dead discrimination. Flow cytometry data were acquired on a BD LSR Fortessa flow cytometer and analyzed using FlowJo software (BD Biosciences). Subsequent statistical analysis and graph generation were performed using GraphPad Prism.

### 4.9 Data Analysis and Statistics

#### 4.9.1 scRNA‐Seq Data Processing and Analysis

We annotated the transcriptomic data using the Scanpy package (v1.10.2). Quality control was applied to filter out cells expressing fewer than 100 genes and genes expressed in fewer than 3 cells. Doublet detection was performed using Scrublet [40], and identified doublets were removed. Next, we selected the top 2000 highly variable genes for dimensionality reduction. These genes were used to compute the neighborhood graph of cells, which was further visualized using UMAP [41]. Finally, we identified cell types such as ‘Mes‐like’, ‘TB‐like’, ‘Fibro’, ‘PSC‐Ect’, ‘PSC‐like’, ‘EC’, ‘Mes‐PS’, and ‘Endoderm’ based on known marker genes. The identified cell types were visualized in UMAP.

#### 4.9.2 RNA Velocity Analysis

To investigate dynamic transcriptional states and infer potential cell fate transitions, we performed RNA velocity analysis using the scVelo (v0.2.4) and cell2fate (v0.1a0) [23] packages. Spliced and unspliced count matrices were obtained from loom files processed by velocyto (v0.17.15) and integrated with cell‐type annotated transcriptomic data using scv.utils.merge(). We applied the Cell2fate_DynamicalModel to reconstruct transcriptional dynamics, automatically determining the number of modules based on data structure. The model was trained using a GPU‐accelerated PyTorch backend with default parameters over multiple iterations until convergence. Velocity‐inferred latent time and module activation states were projected onto the UMAP embedding to visualize developmental trajectories.

We further identified gene modules associated with specific transcriptional programs and computed module‐specific velocity vectors. Genes within each module were ranked by their contribution weights, defined as the proportion of total inferred expression attributable to the module. Transcription factors (TFs) were specifically identified by overlapping the ranked genes with a curated list of human TFs provided in the Cell2fate package. Top features, including these identified TFs, were selected based on this ranking. Gene expression values visualized in UMAP plots were min‐max normalized to a [0, 1] scale to ensure consistent interpretation. Visualizations of velocity streamlines, module activation states, and key gene expression patterns were generated using customized plotting functions based on scVelo and Scanpy.

#### 4.9.3 10x Visium Spatial Transcriptomics Processing

The sequencing data and imaging tiff files of individual 10x Visium slides were processed using SpaceRanger software (version 1.3.1) with the GRCh38 human reference genome. Count matrices were then loaded into the Scanpy package (version 4.1.0) in Python. Spots were selected based on RNA counts and mitochondrial gene percentage. After spot selection, high variable genes (HVGs) were identified and used for principal component analysis (PCA) to reduce dimensionality and perform clustering. The Scanpy adata objects were utilized for downstream analysis individually.

#### 4.9.4 Spatial Transcriptomics Spot Deconvolution

Two packages were utilized for spatial transcriptomics spot deconvolution. The first method applied was SpatialScope, a newly developed statistical method that integrates scRNA‐seq and spatial transcriptomics data to obtain the spatial distribution of the whole transcriptome at the single‐cell resolution. SpatialScope initially performs nuclei segmentation to count and locate nuclei within each spatial spot. It then assigns a cell type to each located nucleus. Finally, by leveraging the paired scRNA‐seq reference data and a learned deep generative model, it decomposes gene expression at each spot into gene expression of the individual cells located within the spot. The RCTD package (version 2.0.0) was also utilized to validate the cell proportion [42]. It first takes the single‐cell reference and predicts the proportion of each cell type in an individual 10x Visium spot.

#### 4.9.5 Cell Interaction within Spatial Transcriptomics Data

Two different packages were utilized for spatial cell‐cell interaction analysis. After obtaining single‐cell resolution spatial data, CellPhoneDB within the Squidpy toolbox was utilized to predict cellular ligand‐receptor interactions [43, 44]. All predicted pairs were pre‐selected based on a p‐value of 0.001. The enriched score and metadata of individual pairs were then examined, and the final results were presented in a bar plot.

#### 4.9.6 Mitochondrial Variant Calling

In the MQuad pipeline, data pre‐processing was performed before mitochondrial variant calling. After getting the fastq files of MAESTER and Visium‐MAESTER data, umitools were first used to generate a whitelist of valid barcodes. The number of cells was estimated by the data. Based on the whitelist of valid barcodes, umitools extracted reads coming from real cells and moved UMIs and barcodes from sequence lines to the headlines. In this step, background reads and barcodes with a low number of reads were filtered. Reads were aligned to the reference genome by STAR using hg38 as the reference genome. UMIs and barcodes were added as tags in the bam file by using pysam. Mitochondrial variants were called by cellsnp‐lite mode 2a. We used a minimum minor allele frequency 0.1 and a minimum aggregated count as a filtering criterion. MQuad [11] was applied to call informative mitochondrial variants for the following analysis.

For the Maegatk pipeline, pre‐processing of the raw sequence files is required before maegatk processing. The maegatk pipeline was promoted as being superior to the previous mgatk tool due to its capability in performing UMI consensus using the additional ‘‐mr’ parameter flag in filtering out reads with less than the minimally specified amount of reads. This is speculated as an important aspect in pre‐filtering mtDNA reads to improve the subsequent variant calling results. However, when comparing the output generated using UMI consensus and without, the distinction is unclear (Figure S5a). The number of variants identified using the same threshold varies for each dataset and does not suggest higher sensitivity after applying a UMI consensus limit of 3 when running maegatk. Yet, there is insufficient data from this study to conclusively dismiss the advantages of the added UMI parameter in maegatk processing, thus further analysis is required to determine the importance of this added parameter when considering using maegatk for MAESTER mtDNA sequencing data processing. Using the maegatk output generated with the suggested minimum 3 UMI reads parameter, cells with more than 1% VAF for any identified variant are selected and subset for the subsequent clonal assignment step.

#### 4.9.7 Clonality Reconstruction

In the MQuad pipeline, clone assignment was performed using vireoSNP [45]. First, we determined several candidate optimal clone numbers by analyzing an elbow plot, following the guidelines from the ‘Mito Clones’ tutorial. Based on this tutorial, we then generated assignment probability plots and mean allele ratio plots, starting with the lowest number of candidate clones. The optimal clone number was selected to maximize clonal resolution while ensuring high‐confidence assignments. Specifically, we retained as many clones as possible, provided that each clone exhibited unique patterns of allele frequency in terms of allele ratios. Additionally, we ensured that each clone had a high assignment probability for its constituent cells. Finally, we used the anno_heat function from vireoSNP to visualize the allele frequency heatmap for each cell.

In the maegatk pipeline, the clValid R package [25] was employed to determine the optimal number of clusters and clustering method based on the variant allele frequency matrix. This package provides statistical validation for clustering and was used here to evaluate various combinations of cluster numbers and clustering methods. Internal validation metrics—such as silhouette width, Dunn index, and connectivity scores—were used to assess and visualize the quality of clonal clustering iterations. For all datasets, hierarchical clustering was identified as the optimal method, with varying numbers of clusters selected based on the validation results. In the next step, hierarchical clustering was performed using the eclust function in R, with Euclidean distance as the metric and the Ward.D method to minimize inter‐cluster variance.

#### 4.9.8 LARRY Lineage Tracing Analysis

LARRY barcode‐based lineage tracing was performed to find the clonal relationships that underpin cellular fate determination. The analytical workflow comprised the following steps: (1) Preprocessing of raw data to eliminate cells with invalid LARRY barcodes, by flowing the quality control framework established by Weinreb et al. [4]; (2) Assignment of clones through the aggregation of barcodes utilizing similarity matrices; (3) Further filtration of LARRY clones containing only a single cell for downstream analysis; (4) Integration with single‐cell RNA sequencing (scRNA‐seq) annotations via the Cospar package (v0.3.1) to establish correlations in lineage fate [6]. A statistical evaluation of clonal biases across cell types was conducted using Fisher's exact test with Benjamini‐Hochberg correction, implemented within the Cospar computational framework. The LARRY barcodes between datasets are non‐overlapping (only 4/7361 overlapped, later excluded). Therefore, lineage tracing analyses were performed independently for D4 and D8. We do not trace clones across time points; each dataset provides a stage‐specific snapshot of clonal dynamics. See Table S1 for details.

#### 4.9.9 Data Integration for Quality Control

To verify the robustness of cell state annotations across experimental batches, we performed a quality control integration of the D4 and D8 datasets. After combining the data and performing PCA, we applied Harmony to adjust the principal components and correct for batch effects. The harmonized components were then used as input to compute the neighbor graph and generate UMAP embeddings for visualization. This analysis confirmed that cell types defined in the time‐point‐specific analyses formed consistent clusters in the integrated space. All subsequent deep‐dive analyses (e.g., RNA velocity, cell2fate module inference, clonal tracing) were therefore performed on the time‐point‐specific datasets to preserve stage‐specific biological signals.

#### 4.9.10 Gene Set Enrichment Analysis (GSEA)

Gene set enrichment analysis for the top transcription factors of each cell2fate module was performed using the GSEApy (v1.1.4) implementation of the Enrichr tool. The analysis was run against the following gene set libraries: GO_Biological_Process_2023, KEGG_2021_Human, and Reactome_2022. Significantly enriched terms, filtered by a false discovery rate (FDR) cutoff of 0.05, were ranked by adjusted *p*‐value. The results were visualized using bar plots to identify key biological processes and pathways associated with each regulatory module.

#### 4.9.11 MAESTER Dataset Processing Pipeline

The MAESTER dataset analysis involved a comprehensive four‐module processing pipeline: (1) Read alignment was performed using Cell Ranger (10x Genomics) with the GRCh38 human reference genome; (2) Variant calling was conducted through cellSNP‐lite (v1.2.0) utilizing parameters (minimum allele frequency [MAF] = 0, minimum count = 1) to enhance variant retention; (3) Informative variant selection was executed via MQuad (v0.1.3) with default thresholds; (4) Clone assignment was implemented using vireoSNP (v2.1.4), which determined optimal clone numbers through elbow plot analysis of clustering stability metrics. Cells lacking variants were filtered out, specifically those without any variants with coverage > = 2 and an alternative allele. Candidate variants are further filtered for variants with allele frequency >0.05 across 95% of cells and less than 5% of cells.

#### 4.9.12 Mapping mtDNA Variants into Spatial Space

The integration of mtDNA variants with spatial information enables the identification of the spatial distribution of mtDNA within the sample. Initially, we map mtDNA variant information into spatial contexts by identifying spatial coordinates using cell barcodes. Following integration, the mtDNA clone information and single‐nucleotide polymorphism (SNP) allele frequencies for each cell are visualized within the spatial framework.

To further analyze mtDNA data, we employ SpatialDE (version 1.1.3), a tool originally designed to identify genes significantly dependent on spatial coordinates. The allele frequencies of mtDNA are normalized to a scale of 100, after which SpatialDE is applied to identify spatially variable mtDNA features. Features with an adjusted Q‐value of less than 0.05 are considered significant.

#### 4.9.13 Integration of mtDNA Variants and Spatial Transcriptomics Data

Spatially resolved transcriptomic data were obtained from our previous study [37]. We selected five HEMOs representing diverse cell types within each orgnoid. For each hemo cell, we gathered single‐cell resolution annotations generated through SpatialScope's deconvolution algorithm. The mtDNA variants for each spot were processed using our MAESTER analysis pipeline. We integrated the mtDNA information for each spot with the cell type percentages to investigate (1) the differences in cell types associated with various mtDNA SNPs and (2) the variations in cell types among different mtDNA clones. Upon identifying distinct cell type distributions for different clones, we further analyzed the cell‐cell interactions within a selected clone. The cell‐cell interaction analysis was performed using CellPhoneDB's permutation testing methodology [43], involving 1,000 permutations and retaining interactions that met dual thresholds: statistical significance (adjusted *p*‐value < 0.01) and biological relevance (mean expression score > 6.5). Directionality assignment for inhibitory interactions was derived from Squidpy's module (v1.6.2), where inhibitory relationships were annotated with negative interaction weights.

#### 4.9.14 Quantitative Spatial Metrics and Statistical Framework

To quantitatively characterize the spatial organization of individual clones within HEMOs, we calculated three complementary spatial metrics for each clone in every organoid replicate. First, the distance to the tissue boundary was computed as the minimum distance from each cell of a clone to the convex hull enclosing the 2D tissue section, with smaller values indicating proximity to the tissue periphery. Second, the radial distance from the centroid was measured as the distance from each cell to the geometric center of the entire HEMO; larger average values for a clone reflect a more peripheral location relative to the organoid core. Third, we assessed the degree of clonal clustering using spatial autocorrelation (Moran's I), where positive values (I > 0) signify that cells of the same clone are spatially aggregated beyond what is expected by chance. These metrics were systematically applied to all three clones across five independent HEMO biological replicates. To determine statistical significance, we performed permutation tests for the observed Moran's I values and used paired t‐tests for comparisons of the distance‐based metrics.

## Author Contributions

Yan Xue, Junhao Su, and Yiming Chao contributed equally to this work. Y. X., J. S., Y. C., J. W.K. H., and R. S. conceived the study. Y. X., L. L., Y. X., T. A., and J. W. conducted experiments and generated the data. J. S. and Y. C. conducted most of the informatics analysis with the guidance of Y. H. and J. W.K. H. . Y. X., X. L., M. K. H., Z. S., J. C., Z. L., C. L., R. L., and K. S. C.C. participated in the informatics analysis. Y. X., J. S., Y. C., J. W.K. H., and R. S. wrote the manuscript. All authors edited the manuscript.

## Conflicts of Interest

The authors declare no Conflicts of Interest.

## Supporting information




**Supporting File 1**: advs73840‐sup‐0001‐SuppMat.pdf.

**Supporting File 2**: advs73840‐sup‐0002‐Table S1.docx.

**Supporting File 3**: advs73840‐sup‐0003‐Table S2.csv.

**Supporting File 4**: advs73840‐sup‐0004‐Movie S1.mp4.

## Data Availability

The data for analysis is available at https://www.ncbi.nlm.nih.gov/bioproject/PRJNA855311 The code for analysis is available at https://github.com/sujunhao/HEMO_maester_spatial.
