# Correction to “Single‐Cell Mitochondrial Lineage Tracing Decodes Fate Decision and Spatial Clonal Architecture in Human Hematopoietic Organoids”

**DOI:** 10.1002/advs.74435

**Published:** 2026-02-19

**Authors:** Yan Xue, Junhao Su, Yiming Chao, Lu Liu, Xinyi Lin, Yang Xiang, Mun Kay Ho, Zezhuo Su, Junyi Chen, Zhuojuan Luo, Chengqi Lin, Ruibang Luo, Theo Aurich, Jianfeng Wu, Kelvin Sin Chi Cheung, Yuanhua Huang, Joshua WK Ho, Ryohichi Sugimura

**Affiliations:** ^1^ School of Biomedical Sciences Li Ka Shing Faculty of Medicine The University of Hong Kong Hong Kong Hong Kong SAR China; ^2^ Laboratory of Data Discovery for Health Limited (D24H) Hong Kong Science Park Hong Kong China; ^3^ Centre for Translational Stem Cell Biology Hong Kong China; ^4^ Department of Orthopaedics and Traumatology School of Clinical Medicine Li Ka Shing Faculty of Medicine The University of Hong Kong Hong Kong China; ^5^ Jiangsu Provincial Key Laboratory of Critical Care Medicine Southeast University Nanjing China; ^6^ Jiangsu Province Hi‐Tech Key Laboratory for Biomedical Research Southeast University Nanjing China; ^7^ Department of Computer Science School of Computing and Data Science The University of Hong Kong Hong Kong Hong Kong SAR China; ^8^ Heidelberg University Hospital Heidelberg Germany; ^9^ Department of Diagnostic Radiology School of Clinical Medicine Li Ka Shing Faculty of Medicine The University of Hong Kong Hong Kong Hong Kong SAR China; ^10^ Department of Statistics and Actuarial Science Faculty of Science The University of Hong Kong Hong Kong Hong Kong SAR China

https://doi.org/10.1002/advs.202518084

This correction pertains to Figure 2 of the above‐referenced manuscript, which was published online on 21 January 2026.

During a post‐publication review, the authors identified an error in the assembly of Figure 2. Specifically, the UMAP plots representing the transcription factors FOXP2 (Module 8), ZNF83 (Module 10), TBX4 (Module 11), and HOXC6 (Module 11) were inadvertently misplaced during the figure compilation process due to a copy‐and‐paste technical error. This resulted in the incorrect arrangement of these specific panels within the figure.

The error was strictly limited to the final figure preparation stage and did not affect any underlying data, analytical workflows, statistical analyses, or other results reported in the study.

The authors have now prepared a corrected version of Figure 2, in which all UMAP plots are accurately placed and correspond correctly to their respective transcription factor modules. The corrected figure is provided with this Correction statement.


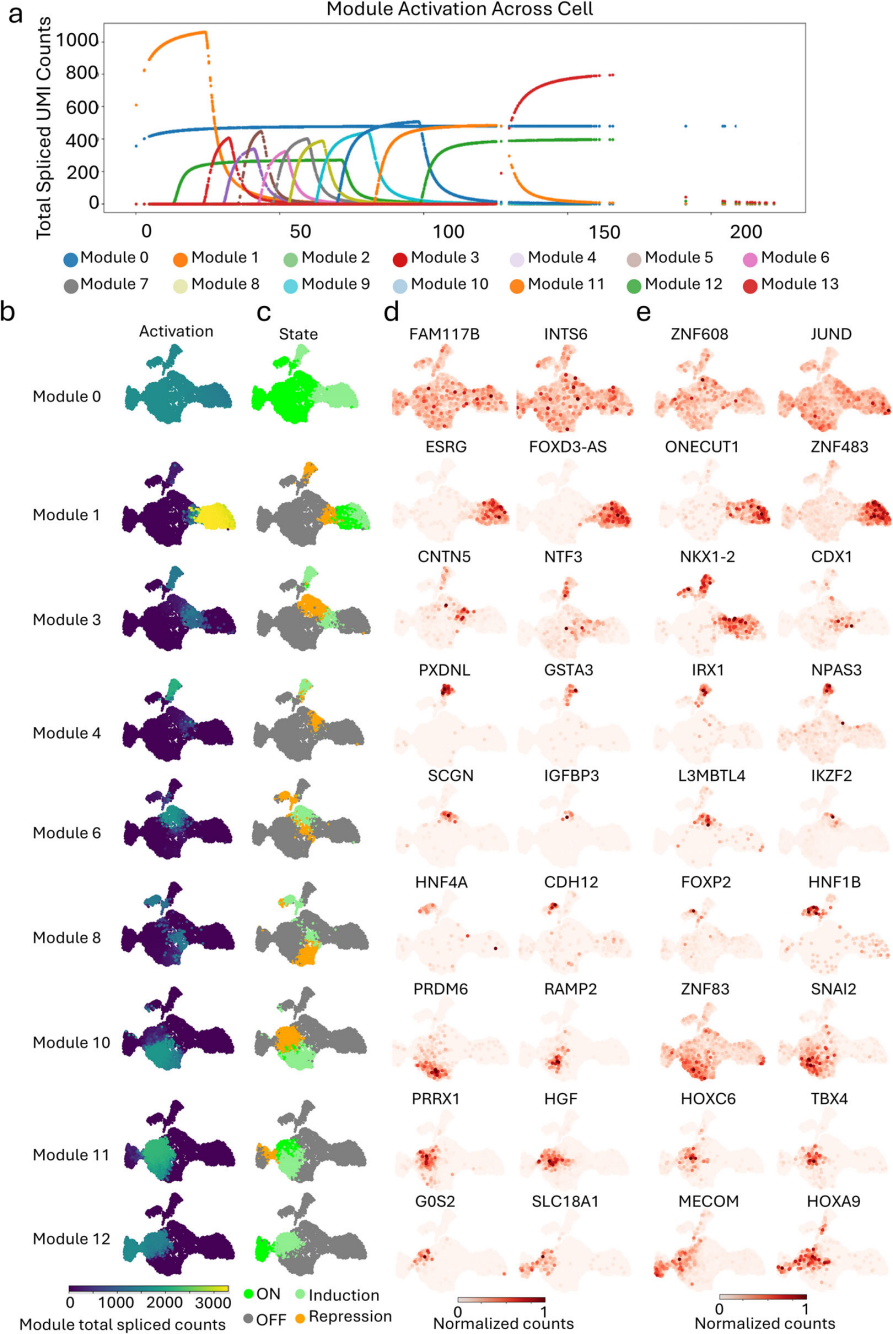


Importantly, this correction does not change any of the scientific interpretations, findings, or conclusions of the manuscript. All results and conclusions remain fully valid and supported by the original data.

We apologize for this error.

