# Supplementary material for: Single‐Cell Mitochondrial Lineage Tracing Decodes Fate Decision and Spatial Clonal Architecture in Human Hematopoietic Organoids
Source: Adv Sci (Weinh). 2026 Jan 21;13(17):e18084. doi: 10.1002/advs.202518084 (PMC13042525; doi:10.1002/advs.202518084)
Supplement: Supplementary file 2 — Supporting File 2: advs73840‐sup‐0002‐Table S1.docx. [file ADVS-13-e18084-s001.docx]

**Supplementary Table 1.** Summary of LARRY barcode statistics from independent D4 and D8 organoid experiments.

| Time Point | Total Clones Detected | Larry Unique barcode | Multi-cell Clones (≥2 cells) | Notes |
| --- | --- | --- | --- | --- |
| D4 | 2,453 | 4,659 | 857 | Independent barcoding experiment; barcodes do not overlap with D8 |
| D8 | 1,391 | 2,698 | 365 | Independent barcoding experiment; barcodes do not overlap with D4 |
